# Neurotrophic effects of intermittent fasting, calorie restriction and exercise: a review and annotated bibliography

**DOI:** 10.3389/fragi.2023.1161814

**Published:** 2023-06-02

**Authors:** Eric Mayor

**Affiliations:** Clinical Psychology and Epidemiology, University of Basel, Basel, Switzerland

**Keywords:** neuroprotection, neurogenesis, synaptic plasticity, intermittent fasting, calorie restriction, caloric restriction mimetics, exercise, brain-derived neurotrophic factor (BDNF)

## Abstract

In the last decades, important progress has been achieved in the understanding of the neurotrophic effects of intermittent fasting (IF), calorie restriction (CR) and exercise. Improved neuroprotection, synaptic plasticity and adult neurogenesis (NSPAN) are essential examples of these neurotrophic effects. The importance in this respect of the metabolic switch from glucose to ketone bodies as cellular fuel has been highlighted. More recently, calorie restriction mimetics (CRMs; resveratrol and other polyphenols in particular) have been investigated thoroughly in relation to NSPAN. In the narrative review sections of this manuscript, recent findings on these essential functions are synthesized and the most important molecules involved are presented. The most researched signaling pathways (PI3K, Akt, mTOR, AMPK, GSK3β, ULK, MAPK, PGC-1α, NF-κB, sirtuins, Notch, Sonic hedgehog and Wnt) and processes (e.g., anti-inflammation, autophagy, apoptosis) that support or thwart neuroprotection, synaptic plasticity and neurogenesis are then briefly presented. This provides an accessible entry point to the literature. In the annotated bibliography section of this contribution, brief summaries are provided of about 30 literature reviews relating to the neurotrophic effects of interest in relation to IF, CR, CRMs and exercise. Most of the selected reviews address these essential functions from the perspective of healthier aging (sometimes discussing epigenetic factors) and the reduction of the risk for neurodegenerative diseases (Alzheimer’s disease, Huntington’s disease, Parkinson’s disease) and depression or the improvement of cognitive function.

## 1 Introduction

Exercise, intermittent fasting and calorie restriction (EIC) have common beneficial effects on energy metabolism and signaling pathways: All three can induce metabolic switching from glucose to ketone bodies (g-to-k) as cellular fuel source when glucose is scarce (Mattson et al., 2018). By lowering of available cellular energy and through the cellular sensing of limited availability of nutrients^1^ which ensues, they can induce the activation or inhibition of several signaling pathways relevant for neuroprotection, synaptic plasticity and neurogenesis (NSPAN) (e.g., Van Praag et al., 2014; Mattson, 2019; García-Rodríguez & Giménez-Cassina, 2021). This can have short-term implications for cognitive performance (e.g., Diano et al., 2006). The benefits of EIC are achieved in part through the influence of hunger hormone ghrelin on glucose homeostasis and the activity of the insulin receptor (Chabot et al., 2014; Bayliss et al., 2016) which is an upstream effector of signaling pathways relevant for NSPAN (Sadria & Layton, 2021). The hormone ghrelin also plays a role in other signaling pathways regulating NSPAN (Diano et al., 2006; Gahette et al., 2011; Bayliss et al., 2016; Buntwal et al., 2019; Davies, 2022). Exerkines can also regulate the activity of these pathways among others (e.g., Chow et al., 2022; Vints et al., 2022; Reddy et al., 2023). Calorie restriction mimetics (CRMs; polyphenols and other sirtuin activators as well as precursors of nicotinamide adenine dinucleotide, in particular) do not rely upon glucose depletion, but they can produce similar effects, including enhanced energy metabolism, decreased oxidative stress and inflammation and enhanced NSPAN and cognition (Testa et al., 2014). The efficacy of CRMs with regards to overall physiological parameters has been questioned by Handschin (2016), but general consensus in the literature points out to the potential of CRMs in the prevention and treatment of neurodegenerative disorders (e.g., Bonkowski & Sinclair, 2016). CRMs are considered promising in this area (e.g., Bonkowski & Sinclair, 2016; Heberden, 2016; Carosi & Sargeant, 2019; Sharman et al., 2019; Almendariz-Palacios et al., 2020; Hofer et al., 2021; Martel et al., 2021; Xue et al., 2021), but the state of current knowledge is insufficient to recommend CRMs for the treatment of individuals presenting with neurodegenerative disorders. For instance, Heger (2017) mentions that only 17 of 45 trials, at that time, show an efficacy of curcumin (in relation to diverse clinical targets). Hofer et al. (2021) provide a summary of human clinical trials interested in various CRMs relating to a diversity of outcomes; Martel et al. (2021) focus on human clinical trials interested in CRMs and the extension of human healthy lifespan, including neuroprotection and neurogenesis, discussing advantages and disadvantages. Treatment should always be provided after sufficient clinical trials have been conducted, but early in the progression of the disease (e.g.,; Carosi & Sargeant, 2019; Sharman et al., 2019).

EIC and CRMs are beneficial in the attenuation of epigenetic changes induced by aging, which are important contributors to neurodegeneration among other risks (Mattson et al., 2018; Madeo et al., 2019; Almendariz-Palacios et al., 2020; Maharajan et al., 2020; Xue et al., 2021). The aims of this article are threefold: A) to discuss the relevance of EIC and CRMs to NSPAN relying upon recent publications, B) to briefly present some of the biochemical processes involved in metabolism, oxidative stress and NSPAN and C) to present a selective annotated bibliography of existing literature reviews (summaries in Appendix) that focus on the ways in which EIC and CRMs can improve NSPAN, cognitive function, and brain resilience (e.g., van Praag et al., 2014).

## 2 The relevance of exercise, calorie restriction (CR) and intermittent fasting (IF) and CRMs in neurogenesis and neuroprotection and other neurotrophic effects

The body can transition from using glucose for energy to using ketone bodies (García-Rodríguez & Giménez-Cassina, 2021; for more details, see section B below): The liver glycogen store can contain up to the equivalent of about 700 calories. It is depleted when energy intake does not occur for a period of 10–12 h at least, or during periods of high energy expenditure (e.g., running for 1 hour following a 4 hours period after the last meal) (Mattson et al., 2018) as glycogen is also stored in the skeletal muscles (Richter & Hargreaves, 2013). EIC can result in the depletion of the glycogen store and thus can activate the g-to-k metabolic switch. The k-to-g metabolic switch occurs when the glycogen store is repleted, after energy intake. The repeated switching between glucose and ketone bodies as main sources of cellular fuel has advantages at the organism level in terms of cellular regeneration and protection, including in the brain in terms of neurogenesis and neuroprotection (Matteson et al., 2018). CRMs such as resveratrol and rapamycin have potential in the prevention and treatment of neurodegenerative diseases (Albani et al., 2010; Baur & Sinclair, 2006; dos Santos et al., 2022; Rege et al., 2014). “[B]oth compounds also show impressive effects in rodent models of age-associated diseases.” (Kaeberlin, 2010, p. 96). This could be because they can modulate cell signaling close to a fasting state (e.g., resveratrol: Chatam et al., 2022; rapamycin; Blagosklonny, 2019). A sustainable alternation of exercise, IF or CR and the use of CRMs might lead to benefits similar to those of the continued reliance upon a single modality of NSPAN intervention. In this contribution, the potential benefits of EIC and CRMs will be dwelved upon, including the metabolic and signaling processes trough which these advantages are achieved.

### 2.1 Neurodegenerative diseases and depression

The large majority of the papers mentioned in this review mention the devastating consequences of aging as one of the motors of research in the neurotrophic effects of EIC and CRMs, including NSPAN. A variety of essential restorative functions are decreased with age, which leads to brain aging (e.g., Zia et al., 2021). In addition, aging increases damage to DNA and tissues, including in neurons, stem cells and their niches, (Maharajan et al., 2020; Brunet et al., 2022). A bibliometric analysis of the literature on cognitive aging is provided by Othman et al. (2022). Some of the consequences of aging are due to epigenic changes in signaling pathways (e.g., Iside et al., 2020; Wang K. et al., 2022). For instance, aging-related impaired AMPK signaling (see below) is related to atherosclerosis and cardiovascular diseases, increased risks of insulin resistance and diabetes, obesity, chronic inflammation, nonalcoholic fatty liver diseases and polycystic ovary syndrome, cancer and neurodegenerative diseases (Yadav et al., 2017). Neurodegenerative diseases share some ethiological processes, such as oxidative stress, post-translational modifications, metabolic changes, cell death mechanisms, disturbed immune response (Fan et al., 2017; Balusu et al., 2023). These factors have (epi)genetic causes and environmental and behavioral causes (Fan et al., 2017).

The most frequent neurodegenerative diseases are Alzheimer’s disease and Parkinson’s disease (McGregor & Nelson, 2019). Alzheimer’s disease is characterized by an aggregation of amyloid beta peptide and accumulation of misfolded tau proteins, which can result from epigenetic dysregulations of cell signaling, including mitochondrial and metabolic disfunctions (Bloom, 2014; Aman et al., 2021; Amorim et al., 2022; Guo et al., 2022; Balusu et al., 2023). Examples of dysregulated processes linked to neurodegenerative impairments in Alzheimer’s disease are endocytosis, proteastasis and phagocytosis in microglia, inflammation and metal ion homeostasis in astrocytes, as well as myelin loss and lipid deposits in oligodendrocytes (Balusu et al., 2023). Parkinsons’ disease is characterized by the presence of Lewy bodies in different areas of the brain and neurodegenerative cell loss, dopaminergic neurons in particular (McGregor & Nelson, 2019; Balusu et al., 2023). Examples of dysregulated processes associated with neurodegeneration in Parkinsons’ disease are: proteostasis, potassium ion transport and phagocytosis in the microglia, heat-shock response and metal ion homeostasis in astrocytes, as well as proteostasis and zinc homeostasis in oligodendrocytes (Balusu et al., 2023).

Major depression is the most important factor associated with disability among all causes (Craske et al., 2017). The search for biomarkers of major depression is challenging due to the heterogeneity of the description of the disorder (e.g., increased appetite/sleep, decreased appetite/sleep; decreased emotionality, increased nervousness; feeling slow, feeling restless); dysregulated cell signaling protein pathways in relation to inflammation and metabolism are candidate biomarkers for major depression (van Haeringen et al., 2022).

Progress stemming from a focus on brain circuits has been disappointing in research, prevention and treatment of neurodegenerative disorders and depression; and some authors point out the relevance of examining the etiology and treatment of neurodegenerative diseases at the molecular and cellular level (Balusu et al., 2023). EIC and CRMs have the potential to maintain healthy cell signaling and restore dysregulated cell signaling (e.g., van Praag, 2008; Spasić et al., 2009; van Praag et al., 2014; Bayliss et al., 2016; Heberden, 2016; Ntsapi & Loos, 2016; Overall et al., 2016; Kallies et al., 2019; Radak et al., 2020; Walsh et al., 2020; Erbaba et al., 2021; Igwe et al., 2021; Ravula et al., 2021; Szwed et al., 2021; Magliulo et al., 2022; Vints et al., 2022). EIC and CRM are discussed next.

### 2.2 Exercise

The American College of Sports Medicine (ACSM, 2013) defines physical exercise as planned voluntary physical activity performed with the aim of maintaining or improving the fitness of the body. There are several types of exercise. Distinctions include: aerobic vs. anaerobic exercise and strength vs. endurance training (ACSM, 2013). In aerobic exercise, the body relies on aerobic metabolism (oxygen provided by pulmonary respiration) to generate the adenosine triphosphate (ATP) necessary for the activity, whereas in anaerobic exercise anaerobic glycolysis is relied upon for ATP production. Strength training aims to increase the force of the muscle. Endurance training aims to increase the potential duration of exertion (ACSM, 2013). While exercise can reduce oxidative stress and chronic inflammation (e.g., Gomez-Cabrera et al., 2008; Beavers et al., 2010), exercise is also a source of reactive oxygen species (ROS) and thereby can increase oxidative stress (Bloomer et al., 2005; He et al., 2016) and the antioxidative benefits of exercise therefore depend upon its duration, type and intensity (Gomez-Cabrera et al., 2008; Fisher-Wellman & Bloomer, 2009). The importance of the redox balance is relation to exercise is discussed in Sutkowy et al. (2021).

Exercise can still provide benefits even when it is oxidative. These benefits include the activation of 5’ adenosine monophosphate-activated protein kinase (AMPK, see below) (Richter & Ruderman, 2009; Richter & Hargreaves, 2013) and of sirtuins (SIRTs) (Radak et al., 2020). In addition to these, the indirect increase in the concentration of brain-derived neurotrophic factor (BDNF) is also a potential consequence of exercise (Leckie et al., 2014). “BDNF mediates beneficial effects of energetic challenges such as vigorous exercise and fasting on cognition, mood, cardiovascular function and peripheral metabolism.” (Marosi & Mattson, 2014, p.89). But these benefits can be balanced by the inflammation stemming from oxidative stress. In the recent years, the important role of exerkines–defined as “any humoral factors secreted into circulation by tissues in response to exercise” (Magliulo et al., 2022, p. 105)—has been highlighted in the explanation of the benefic effects of exercise (e.g., Chow et al., 2022; Magliulo et al., 2022; Vints et al., 2022; Reddy et al., 2023). Vints et al. (2022) discuss the implications of exercises (and exerkines) with regards to synaptic plasticity. A recent meta-analysis (Pillon et al., 2020) has focused on studies relying upon transcriptomic profiling to identify genes of which the expression is modified by exercise in the skeletal muscles. The mechanisms at play in exercise-induced tissue regeneration including in the central nervous system (neurogenesis as well as neuron myelin and axon regeneration for instance) are described by Chen J. et al. (2022). Cortes and De Miguel (2022) highlight the sex differences in CNS responses to exercise.

### 2.3 Intermittent fasting

The overconsumption of food is an issue in modern societies because of its constant availability. Discussing the detrimental effects of overconsumption for cognitive functioning and the damaging implications regarding neurodegenerative diseases, Mattson (2019) specifies that “[t]he underlying molecular mechanisms involve epigenetic modifications (…), that alter the expression of genes involved in neuroplasticity” (p. 204) and lead to “synaptic dysfunction, impaired neurogenesis [and] neuronal degeneration” (p. 205). Nutritional practices that re-establish “adaptive cellular signalling,” such as IF and CR, can mitigate the negative effects of food overconsumption on cognition by enhancing NSPAN (Mattson, 2019).

“Intermittent fasting (IF) is a regimen in which there are repeating cycles of *ad libitum* eating and fasting” (Erbaba et al., 2021, p. 2). Four main forms of IF are frequently studied (in addition to religious forms of fasting): alternate day fasting (important lowering of energy intake every 2 days), whole day fasting (one or 2 days of food abstinence a week) and time restricted feeding (energy intake limited to a time window of 8–12 h, generally) and periodic fasting (were 5 days of *ad libitum* diet are followed by 2 days of fasting) (e.g., Tinsley & La Bounty, 2015; Seidler & Barrow, 2022; Bhoumik et al., 2023; Li et al., 2023). Similar benefits as those of CR (e.g., improved neurogenesis, Baik et al., 2020), which are discussed next, are observed for IF and fasting mimicking diets (Baik et al., 2020; Seidler & Barrow, 2022; Zhao et al., 2022), which consist of limiting the intake of calories and proteins for cycles of a few days to a week (see Seidler & Barrow, 2022). Chen J. et al. (2022) provide a bibliometric analysis on intermittent fasting. Li et al. (2023) provide a balanced view of intermittent fasting (including potential disadvantages) and summaries of processes relating IF to circadian rhythms.

### 2.4 Calorie restriction

CR entails the limitation of energy intake from food—“A rough guideline would be 1,800–2,200 kcal/day for men and 1,600–2,000 calories for women” (Mattson, 2019, p. 8)—for a prolonged period of time (Mattson, 2019). The availability of nutrients (such as the ratio of adenosine monophosphate over ATP; Yadav et al., 2017) regulates several signaling pathways of relevance for NSPAN. Through various mechanisms, CR can delay the cognitive effects of aging (Gillespie et al., 2016). For instance, CR leads to the reduction of oxidative stress (Almendariz-Palacios et al., 2020; Yu Q. et al., 2020), to the proliferation of new neurons (Mattson & Arumugam, 2018; Maharajan et al., 2020), and to a better regulation of autophagy of neurons, i.e., the recycling of the material of deficient or damaged cells which is increased when energy is scarce (in the Appendix, see the summary of Ntsapi & Loos, 2016). Other examples include: the increase of anti-inflammatory hormones, the decrease of chronic inflammation, and the modulation of cell survival (e.g., through apoptosis) (Testa et al., 2014). Some of the signaling pathways involved in these processes and others are described in the next section.

### 2.5 Calorie restriction mimetics

CRMs share some of the benefits of EIC (Testa et al., 2014; Xue et al., 2021). For instance, Bonkowski & Sinclair (2016; see Appendix) discuss the increasing use of nicotinamide adenine dinucleotide precursors and activators of SIRTs in clinical trials in the context of (neurodegenerative) disease prevention and treatment and the promotion of longevity. Several reviews summarized in the Appendix discuss the role of polyphenols in the modulation of synaptic plasticity, increased neuronal proliferation, and the reduction of oxidative stress and of neurotoxicity in particular (e.g., Rendeiro et al., 2015; Moosavi et al., 2016; Zhang et al., 2021). It should be noted that Miller et al. (2011) found that resveratrol and simvastatin did not provide longevity benefits in rodents, whereas rapamycin did. These three CRMs thus impacted longevity differently in Miller et al. (2011) (but see Madeo et al., 2019). Sharman et al. (2019) note a low success rate of phytocomponents (among which nicotinamide adenine dinucleide boosters and sirtuins upstream effectors) in terms of symptom reduction in human trials of Alzheimer’s patients. Importantly, the safety of nicotinamide mononucleotide, a nicontinamide adenine dinucleotide precursor, needs to be further investigated (Nadeeshani et al., 2022).

### 2.6 Neurogenesis

Adult neurogenesis refers to the formation of new neurons in the adult central nervous system (Overall et al., 2016). A description of the different stages of neurogenesis, starting with the stem cell and leading to the mature neuron, is provided by Overall et al. (2016). New neurons are hyperexcitable and less receptive to inhibitory neurotransmitters (e.g., gamma-Aminobutyric acid–GABA), which confers on them more influence than older neurons in a neural circuit (Fares et al., 2019). Adult neurogenesis has been documented in different mammals (Lucassen et al., 2020). Studies on the matter overwhelmingly conclude that neurogenesis occurs in the adult human hippocampus (see Kempermann et al., 2018; Lucassen et al., 2020). The hippocampus fulfills important functions with respect to spatial and episodic memory and is involved in emotional processes (Anacker & Hen, 2017). Neurogenesis in this region–accounting to 700 new neurons every day (2% of the total; Spalding et al., 2013)—might be required for flexible representations and behavior, i.e., in dealing with new situations adaptively (Tuncdemir et al., 2019). Reduced hippocampal neurogenesis is associated with diminished ability to form new memories and the forgetting of old memories (Frankland et al., 2013), whereas increasing hippocampal neurogenesis allows for more flexibility in strategy choice, and can compensate preceding deficiencies in the aging brain (Bergugo-Vega et al., 2020).

Adult neurogenesis can be induced or enhanced through EIC and CRMs, which slows down cognitive decline and can also enhance cognition (e.g., Mattson & Arumugam, 2018; Chow et al., 2022). Overall et al. (2016) describe the different mechanisms through which neurogenesis is promoted by physical activity. Maharajan et al. (2020) and Negredo et al. (2020) discuss the potential of CR in maintaining the health of stem cells and stem cell niche regeneration. Fares et al. (2019) provide a historical perspective on the study of neurogenesis.

### 2.7 Neuroprotection

EIC and CRMs can promote neuroprotection and thereby delay cognitive aging (e.g., Mattson & Arumugam, 2018; Burtcsher et al., 2022; Reddy et al., 2023). This is achieved through several mechanisms. Two are addressed in this paragraph, while others are briefly mentioned in the pages that follow. The first of these mechanisms is the protection of the neuron and of the micro-environment of neurons from oxidative stress (e.g., protection of membrane and DNA integrity; Li et al., 2022; Lüscher et al., 2022). The second related mechanism is the regulation of cell survival (e.g., Ying et al., 2020): In ideal conditions, the survival of healthy cells is promoted, while the survival of senescent and otherwise deficient cells can be impeded through different mechanisms (e.g., autophagy and apoptosis) (e.g., Laberge et al., 2013; Bortner & Cidlowsky, 2020), with neuroprotective effects thereby achieved in the brain (Bhaduri et al., 2023). At the cellular level, different molecules and signaling pathways play a role in determining the fate of the cells, including neurons (e.g., Krieglstein et al., 2002; Fayad et al., 2005; Yamamoto et al., 2007; Huang et al., 2008; Bathina & Das, 2015; Jaworski et al., 2019; Rai et al., 2019; Zhao et al., 2019; Ying et al., 2020; Li et al., 2022; Lüscher et al., 2022; Nordvall et al., 2022). EIC and CRMs can regulate such processes (e.g., Mattson & Arumugam, 2018).

### 2.8 Synaptic plasticity

Synaptic plasticity is also an important neurotrophic effect of EIC and CRMs (e.g., Rendeiro et al., 2015; Seidler & Barrow, 2022; Reddy et al., 2023). The ability of the brain to reorganize following internal and external events is key to learning and adaptive conduct; one such mechanism is synaptic plasticity (McAllister et al., 1999; Bliss et al., 2018; Magee & Grienberger, 2020). Neurotrophins (in particular BDNF, see below in the current section) play an important role in synaptic plasticity as well as synaptic transmission (McAllister et al., 1999; Gómez-Palacio-Schjetnan & Escobar, 2013). Magee and Grienberger (2020) discuss several forms of synaptic plasticity, including the form most commonly studied: Hebbian plasticity (the strength of the synapse depends on how often the presynaptic neuron fires toward the postsynaptic neuron). Abraham (2008) discusses meta-plasticity, which they refer to as the tuning of synapses and neuron networks for plasticity. “Metaplasticity entails a change in the physiological or biochemical state of neurons or synapses that alters their ability to generate synaptic plasticity” (Abraham, 2008, p. 387): Alternations between phases of high stimulation and phases of low stimulation are paradigmatic of the promotion of meta-plasticity, and physical exercise can induce high stimulation.

## 3 Energy metabolism, oxidative stress, neuroprotection, synaptic plasticity andadult neurogenesis: biochemical processes

In this section, several of the processes that are responsible for the effects of EIC and CRMs on NSPAN are discussed. Further, other neurotrophic and protective effects of signaling pathways that are elicited by EIC and CRMs are presented for comprehensiveness.

### 3.1 Energy metabolism

Liver glycogen storage provides the body with continued access to ‘fuel’ as long as the supply lasts through the release of glucose to the bloodstream (Hardie, 2018). Glucose transporters are proteins that mediate the entry of glucose across cell membranes and the blood brain barrier (BBB) (e.g., Simpson et al., 1999; Thorens & Mueckler, 2010). Cellular respiration results in the production of ATP in cell mitochondria (Korla & Mitra, 2014). This process also releases H2O and CO2. Cellular respiration starts with glycolysis (the transformation of glucose to pyruvate, then to acetyl-coenzyme-A; Acetyl-CoA), of which the availability of nicotinamide adenine dinucleotide is an important requirement. It continues with the Krebs cycle, and ends with oxidative phosphorylation—itself involving the electron transport chain (ETC) and chemiosmosis (Zhao et al., 2019).

When energy is necessary in the cell, notably through hydrolysis (the binding of H2O with ATP), ATP is converted to: adenosine diphosphate (ADP) + one phosphate that can be consumed as energy (Yadav et al., 2017). The same can occur for ADP, leading to adenosine monophosphate (AMP) + one phosphate. The ratios ADP:ATP and AMP:ATP are thus indicators of the energy requirements in the cell (Yadav et al., 2017).

Hydrolysis of triglycerides occurs when ADP:ATP or AMP:ATP ratios are high and results in the synthesis of fatty acids, which are then converted to ketone bodies through oxidation to fuel the cells (g-to-k metabolic switching; Newman and Verdin, 2017; Mattson et al., 2018; Martínez-Reyes and Chandel, 2020.

Energy expenditure (e.g., exercise), increases ROS in the organism (He et al., 2016). A major contributor of ROS is the slippage of electrons through the, ETC, in the mitochondria (Zhao et al., 2019; Mazat et al., 2020; Shofield & Schafer, 2021). Proton slippage is partially downregulated by uncoupling proteins (UCPs; Demine and Arnould, 2019; Haas & Barnstable, 2021). Holmström & Finkel (2014) discuss other sources of ROS. The processes of ROS generation and the role of antioxidants and ROS inhibitors are reviewed by Brieger et al. (2012).

Oxidative stress ensues from an elevated production of ROS coupled with insufficient availability of antioxidants (ROS imbalance) (Aon et al., 2010; Shofield & Schafer, 2021). “As signaling molecules, ROS play an important role in cell proliferation, hypoxia adaptation and cell fate determination, but excessive ROS can cause irreversible cell damage and even cell death” (Zhao et al., 2019, p. 3). Oxidative stress more generally can also lead to such negative consequences (Murphy, 2009; Di Meo et al., 2016; Shofield & Schafer, 2021). Zeliger (2013) discusses diseases linked with oxidative stress, their comorbidity and prevention, as well as the Oxidative Stress Index questionnaire, which permits to assess the potential future onset of non-communitive chronic diseases and a list of environmental sources of ROS.

A moderate ROS is ideal for the endocrine, immune and cognitive functions (Brieger et al., 2012) and thus both low and high ROS are related to metabolic disfunctions and diseases (Brieger et al., 2012). Importantly, an elevated ROS is associated with a range of neurodegenerative diseases (Singh et al., 2019). Dunn et al. (2015) present the anti-microbial function of ROS. Holmström & Finkel (2014) discuss redox signaling. Chun et al. (2010) discuss the antioxidant intake of Americans from several sources.

Both high AMP:ATP ratio and high ROS are sensed by the 5’ adenosine monophosphate-activated protein kinase (AMPK) and trigger its signaling (e.g., Rabinovitch et al., 2017; Hinchy et al., 2018). Different protective mechanisms are thereby indirectly activated by increased ROS and involve SIRTs, peroxisome proliferator-activated receptor-γ coactivator (PGC)-1α, mammalian target of rapamycin (mTOR), Unc-51 Like autophagy activating Kinase 1 (ULK1), transcription factors forkhead box O (FOXO) and p53. Promyelocytic leukemia (PML) is a sensor of ROS; when ROS is sensed, it activates p53 (Niwa-Kawakita et al., 2017). P53 is an inhibitor of cell growth and propagation and is involved in tumor suppression (Budanov & Karin, 2008; Aubrey et al., 2018). It is involved in neuroprotection by regulating neuronal apoptosis (Culmsee & Mattson, 2005). Maor-Nof et al. (2021) note that p53 is linked with neurodegeneration.

### 3.2 Nucleotides, metabolites and neurotrophic factors

#### 3.2.1 Nicotinamide adenine dinucleotide

NAD+ is involved in DNA repair and mitophagy (the process of recycling of non-essential or damaged mitochondria; Schofield & Schafer, 2021) as it is required for SIRT1 activation (Fang & Bohr, 2017; Chen et al., 2020). NAD+ participates in glycolysis and is reduced to NADH in the process (Ying et al., 2020; Navas & Carnero, 2021). As mentioned above, a high level of ROS is an important threat to mitochondrial and cellular integrity. Increased NAD+ can upregulate immunity, inflammation and DNA repair through the activation of NAD+ dependent enzymes such as SIRTs and poly(ADP-ribose) polymerases (PARPs) (Xie et al., 2020; Yang et al., 2020; Navas & Carnero, 2021). Although it has been reported that NAD+ acts as a proinflammatory cytokine (e.g., Xie et al., 2020; Navas & Carnero, 2021), it has also been shown that NAD+ and its precursors can regulate chronic inflammation and reduce ROS, and, thereby, inflammation (Covarrubias et al., 2021; Xie et al., 2020). NAD+ also modulates the effect of different enzymes, facilitates energy metabolism and helps in the synchronization of the circadian clock (Xie et al., 2020). NAD+ and NADH also play important roles in cell survival and death (Yang et al., 2020), including through their role in ADP-ribosylation (Li et al., 2022; Lüscher et al., 2022). Aging is related to NAD+ deficiency and such deficiency is related to accelerated aging (e.g., increased mitochondrial disfunctions; Xie et al., 2020). This constitutes a vicious circle–which can be thwarted for instance by supplementation of NAD+ precursors (Bonkowski & Sinclair, 2016) and the upregulation of AMPK signaling through exercise (Richter & Hargreaves, 2013) and calorie restriction (Mattson & Arumugam, 2018). Consequences include disturbed circadian rhythms, increased inflammation, poor immunity, hampered DNA repair, and increased risk of cancer, which is aggravated by high levels of ROS (Xie et al., 2020; Navas & Carnero, 2021).

Three pathways are at play in the synthesis of NAD+: the *de novo*, Preiss-Handler and Salvage pathways (Lautrup et al., 2019; Xie et al., 2020; Ying et al., 2020). Synthesis of NAD+ through the *de novo* pathway has neuroprotective and neurotoxic effects (Lautrup et al., 2019). It “starts with the catabolism of the amino acid tryptophan that is converted *via* two steps to the intermediate kynurenine, which can generate NAD+, kynurenic acid, or xanthurenic acid.” (Lautrup et al., 2019, p. 630). NAD+ is synthesized from nicotinic acid (NA) through the Preiss-Handler pathway and from nicotinamide riboside (NR) and the reuse of nicotinamide (NAM) through the Salvage pathway (Lautrup et al., 2019; Xie et al., 2020; Ying et al., 2020). Both the Preiss-Handler and Salvage pathways rely upon the intermediary synthesis of nicotinic acid mononucleotide (NAMN, Lautrup et al., 2019; Xie et al., 2020; Ying et al., 2020). It is worth noting that “ATP may be consumed to replete NAD+ when NAD+ is selectively depleted by enzymes such as PARP-1; *vice versa*, NAD+ may also be consumed to regenerate ATP under the pathological conditions when ATP is selectively depleted.” (Ying et al., 2020). The therapeutic potential of nicotinamide riboside supplementation is examined by Mehmel et al. (2020).

#### 3.2.2 Ketone bodies

Fatty acids are synthesized (through hydrolysis of triglycerides) when glucose is lacking, for instance following EIC, and then converted into the three ketone bodies: β-hydroxy-butyrate (BHB), acetone (Ace) and acetoacetic acid (also referred to as acetoacetate, AcAc) (Newman & Verdin, 2017). Ketone bodies are then distributed to the cells of the organs, including the brain, through their release in blood circulation. Ketone bodies can be used as alternate fuel sources to glucose: In the cell, they are catabolized into Acetyl-CoA (Newman & Verdin, 2017). Acetyl-CoA is then converted to ATP as a result of the Krebs cycle (for details, see Martínez-Reyes & Chandel, 2020). Ketone bodies also have regulatory roles, such as the response to oxidative stress, cell excitability, the regulation of protein expression, and signaling more generally in and out of the brain (see Koppel & Swerdlow, 2018; García-Rodríguez & Giménez-Cassina, 2021). There is evidence that increases in ketone bodies, BHB in particular, result in neuroprotection (e.g., Kolb et al., 2021). BHB can be transported across the BBB by monocarboxylic acid transporters (MCT). The amount of MCT regulates BHB in the brain, where it is responsible for a variety of actions (Newman & Verdin, 2017). Some of these are briefly mentioned: An important role of BHB is the upregulation, through activation of NF-κB, of the hippocampal expression of BDNF in case of either scarce or sufficient glucose supply (Hu et al., 2018; Mattson & Arumugam, 2018). In other words, BHB increases BDNF even in the absence of g-to-k metabolic switching. BHB affects sympathetic tone through inhibition of the free fatty acid receptor 3 (Newman & Verdin, 2017). It upregulates gene expression and suppresses oxidative stress by inhibiting class I histone deacetylases (HDAC; Newman & Verdin, 2017; Shimazu et al., 2013). It appears BHB is capable of stimulating PGC-1α (mitochondrial biogenesis and mitophagy) and FOXO1 (metabolic regulation, reduced sympathetic tone, apoptosis; Santo & Paik, 2018) without AMPK modulation through the Phosphoinositide 3-kinase–Protein kinase B (PI3K/Akt) pathway (Kim et al., 2019). Through the activation of macrophages, BHB and nicotinic acid (NA), activate the hydroxy-carboxylic acid receptor 2 (HCAR2) which induce neuroprotection through the reduction of neuroinflammation and also regulates lipid metabolism (Rahman et al., 2014; Newman & Verdin, 2017). BHB is also involved in synaptic plasticity (Mattson & Arumugam, 2018) and GABAergic regulation (Newman & Verdin, 2017) and plays a major role in cognition and health in general (Cavaleri & Bashar, 2018).

#### 3.2.3 Brain-derived neurotrophic factor (BDNF)

BDNF, a member of the neurotrophins family, is a main protagonist in NSPAN that can be increased through exercise (Zhao et al., 2008; Bathina & Das, 2015; Kallies et al., 2019). Neurotrophins are a family of proteins that regulate neural function, growth and survival, and neuroplasticity through various signaling pathways (Huang et al., 2008; Bathina & Das, 2015). There are four neurotrophins: nerve growth factor (NGF), neurotrophin-3 (NT-3), neurotrophin-4 (NT-4) and BDNF. The role played by BDNF in regulating neurophysiological processes, modulating synaptic interactions, neuroplasticity and neuroprotection has been extensively studied (Kowiański et al., 2018). Such functions are mediated by several downstream effectors, including the Trk family of tyrosine kinase family of receptors (Foltran & Diaz, 2016). Activation of the tyrosine receptor kinase B (TrkB) kinase promotes signaling along two pathways: PI3K/Akt and Raf/MEK/ERK (Foltran & Diaz, 2016). A few examples of the more specific roles of BDNF are now provided. Research has found that BDNF increases axonal arborization through tyrosine receptor kinase B (TrkB) signaling, a process which is regulated by the phosphitylation of the mitogen-activated protein kinases (MAPK; Cheng et al., 2011; Jeanneteau et al., 2010). TrkB is further involved in neural plasticity (Johnstone & Mobley, 2020). BDNF also increases the activation of neuronal stathmins (Cardinaux et al., 1997). Stathmins induce plasticity, the generation of new neurons and their development (Chauvin & Sobel, 2015). It is notable that BDNF also upregulates mTORC1, which can be detrimental; but this action is inhibited by AMPK and its effect on SIRT signaling through the upregulation of NAD+ metabolism (Ishizuka et al., 2013; Sadria & Layton, 2021). Reduced levels of BDNF are associated with neurodegenerative diseases (for reviews, see: Bathina & Das, 2015; Autry & Monteggia, 2012—who mention its relation with neurotransmitter serotonin). In the body and in the brain, BDNF is also involved in energy homeostasis and regulation of the parasympathetic tone (Marosi & Mattson, 2014).

Interventions targeting neurotrophins, including BDNF are discussed by Nordvall et al. (2022): agonists of TrkB and BDNF are explored in such interventions, e.g.,: steroid derivatives, “gambogic amide, asiaticoside, and sarcodonin” (p. 3) as well as 7,8-dihydroxy flavone (a polyphenol; see Emili et al., 2022). Several of which did not show robust effectiveness, which questions their validity (Nordvall et al., 2022).

### 3.3 Protein and lipid kinases involved in NSPAN

Kinases are enzymes which add a phosphor group to their target when activated (phosphorylation, Taylor & Kornev, 2011). In this sub-section, several protein and lipid kinases involved in NSPAN are briefly presented. The main actions of these effectors are summarized in Table 1.

**TABLE 1:** Several signaling proteins involved in NSPAN.

Effector	Type	Main actions
PI3K	Lipid kinases	Cell proliferation, autophagy, synaptogenesis
Akt	Protein kinases	Cell proliferation, apoptosis, insulin signaling, protein synthesis
mTOR	Protein kinases	Cell proliferation, protein synthesis, autophagy, immune function
AMPK	Protein kinases	Cellular metabolism, nutrient sensing
GSK3β	Protein kinases	Glycogen metabolism, cell cycle regulation, cell division, gene transcription, insulin signaling
ULK	Protein kinases	Autophagy, mitophagy
MAPK	Protein kinases	Cell proliferation and differentiation, immune responses and inflammation, axon arborization
PGC-1alpha	Transcription coactivators	Regulation of oxidative stress, inflammation, mitochondrial biogenesis and mitophagy
NF-κB	Transcription factor	Metabolism, immunity, inflammation, protein synthesis, cell survival, response to tumor microenvironment
Sirtuins	Protein deacetylases	Gluconeogenesis, lipid metabolism, mitochondrial respiration, cell cycle control, gene expression, apoptosis, inflammation, insulin secretion, neuroprotection, axonal protection, DNA repair
Notch	Transmembrane proteins	Cell fate, development, and differentiation, regulation and plasticity of neural stem cells
Sonic hedgehog	Morphogens	Cell proliferation and differentiation, brain development and neural stem cell maintenance and proliferation
Wnt	Endoplasmic reticulum proteins	Cell proliferation, differentiation, and survival, differentiation of neuronal precursor cells, presynaptic and postsynaptic assembly
PARPs	Nuclear enzymes	Transcriptional regulation, DNA repair, Cell survival and death

*Note*. This table has been created with the help of GPT-3.5 and its content thoroughly verified.

#### 3.3.1 Phosphoinositide 3 (PI3K)

IF and CR are involved in the improvement of mitochondrial function through the regulation of PI3K and its effectors (Lu et al., 2019; Zhao et al., 2022). PI3K dysregulation is implicated in neurodegenerative diseases such as AD (Kumar & Bansal, 2022), which has been associated with increased chronic microglia activation following such dysregulation (Chu et al., 2021): “considerable evidence suggests that chronically activated microglia continually secrete neurotoxic molecules to sustain neuroinflammation” (p. 3). Further, PI3K is involved in the regulation of autophagy, cellular growth and survival and synaptogenesis, notably through its direct or indirect influence on Akt, mTOR (PI3K/Akt/mTOR pathway, Ersahin et al., 2015) and ERK (Carracedo & Pandolfi, 2008; Cuesto et al., 2011; Chen et al., 2013; Rai et al., 2019; Sadria & Layton, 2021). Key activators of PI3K include insulin/insulin growth factor 1 (IGF-1), the tyrosine receptor kinase B (TrkB), and BHB (Foltran & Diaz, 2016; Kim et al., 2019; Sadria & Layton, 2021). PI3K is an upstream regulator of Ras (Bathina & Das, 2015), which itself regulates Raf, the entry point of the Raf/MEK/ERK pathway (Peyssonnaux & Eychène, 2001).

PI3K is upregulated by insulin receptor activation, IGF-1 and TrkB activity in particular (Foltran & Diaz, 2016; Sadria & Layton, 2021). Fakhri et al. (2021) examine the use of natural molecules to reduce the activity of the PI3K/Akt/mTOR signaling pathway. A history of research on PI3K is provided by Vanhaesebroeck et al. (2012) and a bibliometric analysis of the literature on PI3K by Ho & Hartley (2020).

#### 3.3.2 Protein kinase B (Akt)

Akt dysregulation is implicated in neurodegenerative diseases such as AD (Kumar & Bansal, 2022). Akt is involved in neuroprotection in several ways as: up or downregulation of the functions of other proteins responsible for apoptosis (programmed death of deficient cells), and survival and proliferation of cells–including autophagy, notably neurons and the regulation of neuronal toxicity (Fayad et al., 2005; Chen et al., 2013; Rai et al., 2019). Examples include the downregulation of AMPK–through a decrease in the AMP:ATP ratio and the upregulation of mTOR (Hahn-Windgassen et al., 2005). Fayad et al. (2005) mention that other pathways may also regulate pathways modulated by Akt. Akt also phosphorylates FOXO and the tuberous sclerosis complex (TSC1 and TSC2, important regulators of mTOR; Huang et al., 2008; Sadria & Layton, 2021) and regulates the anti-apoptotic and pro-survival extracellular signal-regulated kinases (ERK) (Rai et al., 2019). Vadlakonda et al. (2013) discuss the interplay (‘interactions and feedback loops’) between Akt and mTOR and the mediating role of AMPK and FOXO in particular.

The Insulin/insulin growth factor 1 (IGF-1) pathway activates phosphoinositide-dependent kinase-1 which activates Akt (Sadria & Layton, 2021). Akt activation can also be promoted through PI3K and mTORC2 (Huang et al., 2008; Vadlakonda et al., 2013; Esrahin et al., 2015; Sadria & Layton, 2021).

#### 3.3.3 Mammalian target of rapamycin (mTOR)

mTOR is related to two complexes: mTORC1 and mTORC2. The TSC1-TSC2 complex downregulates mTORC1 and upregulate mTORC2 (Huang et al., 2008). Neurodegenerative diseases are a potential consequence of chronic activation of mTORC1 (Sardia & Layton, 2021). mTOR is inhibited by CR and IF due to their effect on AMPK (Zhao et al., 2022). While exercise has the potential to increase mTOR activation due to the activation of Akt, “far from increasing mTORC1 activity, during running, the high amounts of metabolic stress induced by endurance exercise can decrease mTORC1 activity. This decrease in mTORC1 activity following running may be the result of the activation of the 5′-adenosine monophosphate activated protein kinase (AMPK)” (Watson & Baar, 2014, p. 134). mTOR is both a downstream and upstream effector of Akt. mTOR is involved in sensing “nutrient levels, the presence of growth factors, and the cellular energy status and mediates several catabolic and anabolic processes to maintain metabolism and cell growth” (Ersahin et al., 2015, p. 1947). Specifically, mTOR participates in autophagy (the recycling of cell materials and potentially cell death), the synthesis of proteins and nucleotides as well as glucose, mitochondrial and lipids metabolism (Chen et al., 2013; Liu & Sabatini, 2020; Szwed et al., 2021). When mTORC1 is activated chronically (and potentially in case of severe and prolongued acute activation), the risk of cancer is increased (Sadria & Layton, 2021).

mTORC1 can be directly inhibited by rapamycin (Szwed et al., 2021). It is less active when amino acids are low (Szwed et al., 2021). In particular, its activation results from the binding of arginine with Cytosolic arginine sensor for mTORC1 subunit 1 (CASTOR1) (Budanov & Karin, 2008; Chantranupong et al., 2016; Szwed et al., 2021) and leucine with SESN 2 (Budanov & Karin, 2008; Szwed et al., 2021; Chen Y. et al., 2022). The phosphorylation of ULK1 by AMPK downregulates mTORC1, while the phosphatidylinositol 3-kinase/Akt pathway upregulates mTORC1 (Sadria & Layton, 2021; Szwed et al., 2021). Unlike mTORC1, mTORC2 can not be inhibited by rapamycin directly (Szwed et al., 2021). PI3K activates mTORC2 (Sadria & Layton, 2021). mTORC2 promotes Akt activation through different means (Huang et al., 2008; Esrahin et al., 2015; Sadria & Layton, 2021).

Caron et al. (2010) provide a summary of the upstream and downstream effectors of mTOR. A bibliometric analysis of the literature on mTOR is provided by Li et al. (2022).

#### 3.3.4 5’ adenosine monophosphate-activated protein kinase (AMPK)

AMPK is a considered target in anti-aging approaches aiming at neuroprotection, including the prevention and the alleviation of neurodegenerative diseases (e.g., Bayliss et al., 2016). Primary functions of AMPK are the sensing of cellular energy (e.g., high AMP:ATP ratio, low ATP), and the metabolic regulation that ensues; as well as the regulation of mitochondrial ROS (Rabinovitch et al., 2017; Zhao et al., 2019). Other important functions include a) the subsequent increase or decrease in the oxidized form of nicotinamide adenine dinucleotide (NAD+) and sirtuin-1 (SIRT1, Cantó et al., 2009), b) the down or upregulation of pathways that consume or produce ATP (Cantó et al., 2009) and c) mitochondrial biogenesis (Cantó et al., 2009), neuron cell fate (Spasić et al., 2009). The role of AMPK in cellular metabolism (e.g., fatty acid synthesis, protein synthesis, glycolysis, glucose transport, endothelial nitride oxide synthase) is discussed by Yadav et al. (2017). Importantly, with the result of restoring energy balance in the cell, AMPK upregulates PGC-1α, phosphorizes ULK1 and tuberous sclerosis complex (TSC) 2 and thereby suppresses mTOR signaling (Rabinovitch et al., 2017; Sadria & Layton, 2021), an action which requires the activation of protein kinase B (Akt, Alessi et al., 2006); and SIRT directly (Yadav et al., 2017) and indirectly through increasing NAD+ (Tang, 2016). AMPK serves several other functions, including, among others, the regulation of autophagy (Karabiyik et al., 2021; Penugurti et al., 2022).

In addition to regulation related to energy sensing, AMPK activity is inhibited by ULK1 (Sadria & Layton, 2021) and is activated by cellular stress, leptin/adiponectin, fasting and exercise (Richter & Hargreaves, 2013; Richter & Hargreaves, 2013; Yadav et al., 2017; Hinchy et al., 2018; Chen J. et al., 2022). P53 activates AMPK through Sestrin (SESN) 1 and SESN 2 (Budanov & Karin, 2008). SESNs are stress sensing proteins that participate in cellular homeostasis through the regulation of AMPK and mTOR and play an essential role in disease prevention (Chen Y. et al., 2022). p53 is activated as well by the tumour suppressor serine–threonine kinase liver kinase B1 (LKB1, Alessi et al., 2006; Shackelford & Shaw, 2009), which is also required for AMPK downregulation of mTOR signaling (Shaw, 2009). A bibliometric analysis of the literature on AMPK is provided by Lyu et al. (2022).

#### 3.3.5 Gylcogen synthase Kinase-3β (GSK3β)

The neurotrophic actions of GSK3β are mainy related to its role in the regulation of synaptic plasticity, synaptogenesis and neuronal proliferation and migration (Cuesto et al., 2015; Jaworski et al., 2019). GSK3β is also involved in cell survival and proliferation, metabolism and neuronal function (Jaworski et al., 2019). GSK3β plays an important role in the regulation of circadian rhythms through the activation and inactivation of nuclear factor erythroid 2-related factor 2 (Nrf2), which is also activated by oxidative stress (Shilovsky et al., 2021). Nrf2 upregulates stress adaptation through inhibition of inflammatory processes and ROS reduction (Shilovsky et al., 2021). Dysregulation of GSK3β has implications for neurodegenerative diseases and major depression (Jaworksi et al., 2019). GSK3β is downregulated by phosphorylation (e.g., by Akt) and is activated by dephosphorization (e.g., by serine/threonine protein phosphatase 1) (Jaworski et al., 2019). IGF-1 upregulates PI3K and Akt and downregulates GSK3β (Duarte et al., 2008). Mutations along several pathways (PI3K, hedgehog, Notch, Wnt) are related to alterations in GSK3β activity (McCubrey et al., 2016). GSK3β phosphorylates β-catenin (Hermida et al., 2017), which is essential in the canonical actions of Wnt (Arredondo et al., 2020).

#### 3.3.6 Unc-51 like autophagy activating kinase (ULK)

The ULK complex is composed of ULK1 and ULK2. ULK1 is involved in autophagy (recycling of cell materials) in conditions of cellular stress, which can affect the functioning of the cells and their survival or death (Nie et al., 2016). The ULK complex is also involved in mitophagy, which is the quality control process of the mitochondria with functions such as the “degradation of damaged and superfluous mitochondria, prevent[ion of] detrimental effects [of ROS and mitochondrial disfunction] and reinstat [ion of] cellular homeostasis in response to stress” (Chen et al., 2020, p. 1). Disfunctions in autophagy and mitophagy are importantly linked to neurodegenerative disorders (e.g., Chen et al., 2020). ULK is upregulated by AMPK and inhibited by mTOR (Sadria & Layton, 2021; Yu et al., 2021).

#### 3.3.7 Mitogen-activated protein kinases (MAPK) and signal cascades

MAPK signal cascades play an essential role in the expression of some genes, immunity, cell differentiation and proliferation and are also involved in regulating neuronal inflammation and axon arborization (Peyssonnaux & Eychène, 2001; Wancket et al., 2012; Ersahin et al., 2015). Among those, an important signal cascade is the Raf/MEK/ERK pathway (Peyssonnaux & Eychène, 2001). Raf/MEK/ERK is also involved in cellular apoptosis and thereby regulates cell survival (Peyssonnaux & Eychène, 2001). This signaling pathway is complementary to the PI3K/Akt/mTOR pathway (Ersahin et al., 2015).

However, “MAPKs are implicated in the pathogenesis of many conditions” (Wancket et al., 2012, p. 244). MAPK phosphatases (MKPs) downregulate MAPKs and MAPKs increase the activity of MKPs (Wancket et al., 2012) this feedback loop allows for the beneficial functions of MAPKs to be achieved, while keeping their activity under control. Ras, and Raf indirectly, are upregulated by PI3K leading to the activation of some MAPKs (e.g., ERK; Peyssonnaux & Eychène, 2001). “On the one hand, hypoglycemia [e.g., which can be achieved through EIC] decreases anabolic hormones (e.g., insulin, GH, and IGF1), as well as sex and thyroid hormones, increases the expression of the catabolic cortisol, and subsequently inhibits the MAPK pathway (i.e., RAS/RAF/MEK/ERK) and the PI3K/Akt/mTOR pathway. On the other hand, the inhibition of ERK avoids mTOR activation and subsequently induces autophagy activity, which contributes to the suppression of inflammation by downregulation of both IFN [i.e., interferons] and proinflammatory cytokine responses.” (Kökten et al., 2021, p. 1561).

### 3.4 Other signaling proteins involved in NSPAN

Other signaling proteins which play an important role in NSPAN are presented here. Their main actions are summarized in Table 1.

#### 3.4.1 Peroxisome proliferator-activated receptor-γ coactivator (PGC-1α)

Exercise, calorie restriction and intermittent fasting, as well as exposure to cold temperature, upregulates PGC-1α (Anderson & Prolla, 2009; Vernier & Giguère, 2021; Chen J. et al., 2022). PGC-1*α* plays a major role in a reducing oxidative stress and inflammation and in regulating mitochondrial biogenesis and mitophagy (Tang, 2016; Rius-Pérez et al., 2020). It is also involved in fatty acid oxidation, glucogenesis and energy metabolism (regulation of the Krebs cycle and of ATP production) (Rius-Pérez et al., 2020). “Dysregulation of PGC-1α alters redox homeostasis in cells and exacerbates inflammatory response, which is commonly accompanied by metabolic disturbances.” (Rius-Pérez et al., 2020, p. 1). Dysregulation of PGC-1α accrues with aging, and anti-aging intervention have been documented to target PGC-1α as to limit the impact of mitochondrial decline including on the nervous system (Anderson & Prolla, 2009; Vernier & Giguère, 2021).

PGC-1α is mainly regulated by AMPK (positively), Akt (negatively) and glycogen synthase kinase 3β (GSK3β; negatively) (Rius-Pérez et al., 2020; Chen J. et al., 2022) as well as indirectly by SIRT (Cantó et al., 2013). BHB can upregulate PGC-1α (Kim et al., 2019).

#### 3.4.2 Nuclear factor κB (NF-κB)

Moderate exercise might inhibit NF-κB, while resistance exercise activates it (Vella et al., 2012; Liu et al., 2017). NF-κB is an important regulator of the cell cycle (thereby affecting cell fate), immunity and inflammation (Liu et al., 2017; Mattson & Arumugam, 2018). Such functions of NF-κB are relevant for neurodegeneration (Yu H. et al., 2020). In particular, NF-κB chronic activation has been implicated in the pejoration of symptoms of Alzheimer’s disease and neuroinflammation—a risk factor for neurodegenerative diseases and depression (Dolatschahi et al., 2021; Capece et al., 2022); and NF-κB is involved in contradictory functions such as cell survival and neurodegeneration (Mincheva-Tasheva et al., 2013): “the same stimulus produces opposite responses by activating NF-κB in different cell types” (p. 187). Some of the upstream effectors of NF-κB are: lactate, succinate, BHB, (un)saturated fatty acids) (a more complete list is provided by Capece et al., 2022). NF-κB is also indirectly activated by ROS (Liu et al., 2017).

#### 3.4.3 Sirtuins (SIRT)

SIRTs are a class of histone deacetylases (Carafa et al., 2016). “The seven members of this family of enzymes are considered potential targets for the treatment of human pathologies including neurodegenerative diseases, cardiovascular diseases, and cancer.” Carafa et al., 2016, p. 1). The availability of NAD+ is required for the activation of all 7 SIRTs (Cantó et al., 2009; Tang, 2016; Elkhwanky & Hakkola, 2018; Fagerli et al., 2022). Such requirement fulfills energy sensing functions: Thereby, SIRTs can optimally respond to cellular energy requirements (Elkhwanky & Hakkola, 2018). SIRT1, SIRT6 and SIRT7 are present in the cell nucleus, whereas SIRT2 is present in cytosol and SIRT3, SIRT4, and SIRT5 are present in the mitochondria (Elkhwanky & Hakkola, 2018; Fargeli et al., 2022). Morris (2013; focus on aging) and Yamamoto et al. (2007) addresses in detail the functions of SIRTs, notably: gluconeogenesis, lipid metabolism, mitochondrial respiration, cellular proliferation and differentiation, cell cycle control, gene expression, apoptosis, inflammation, insulin secretion, neuroprotection and axonal protection, DNA repair. Elkhwanky & Hakkola (2018) discuss the important role of extranuclear SIRTs. SIRT1, of which the activity has been in particular associated with increased longevity (for details, see Tang, 2016) has been the focus of most research on SIRTs. In what follows, the activity of SIRTs is discussed in very general terms, as the signaling of SIRTs is especially complex and would require much space (but see Cantó et al., 2009; 2013; Tang, 2016; Elkhwanky & Hakkola, 2018; Fagerli et al., 2022). Specific mentions of SIRT signaling are found in the other sections.

Increased activity of SIRTs (e.g., by AMPK, or indirectly by exercise, CR or CRMs; e.g., Chen J. et al., 2022) is related to energy homeostasis (e.g., regulation of the Krebs cycle and oxidative phosphorylation, triggering of glucose uptake, fatty acid oxidation and ketogenesis), mitochondrial homeostasis and integrity (notably: reduction of ROS and DNA repair through activation of PARPs, see below), and NSPAN (regulation of synaptic plasticity; mitochondrial biogenesis in neurons importantly; reduction of ROS) (Elkhwanky & Hakkola, 2018; Fagerli et al., 2022). Some of the functions in which SIRTs are indirectly involved in rely upon ADP-ribosylation signaling (see below) in which PARPs play a major role (Hottiger, 2015). A higher risk of neurodegenerative diseases ensues the decreased activity of SIRTs, such as mitochondrial and cardio-vascular diseases, and neurodegenerative diseases (Elkhwanky & Hakkola, 2018; Jiao & Gong, 2020; He et al., 2022). Importantly, SIRTs can activate LKB1, which in turn can activate AMPK and downregulate mTOR (Woods et al., 2003; Shaw, 2009; Sadria & Layton, 2021) and regulate PGC-1α (Cantó et al., 2013). The modulation of SIRTs, for instance using resveratrol among other SIRT activators, is a promising prevention and treatment target for neurodegenerative disorders and cancer (Carafa et al., 2016; Cao et al., 2018). Leite et al. (2022) discuss the relevance of targeting SIRTs pharmacologically for the prevention and treatment of neurodegenerative disorders in particular. Fang et al. (2019) discuss the role of SIRTs in the regulation of stem cells.

#### 3.4.4 Sonic hedgehog (Shh)

A recurrent finding has been that signaling pathways essential to the brain development are also active in neurogenesis and synaptic plasticity (Yao et al., 2016). This is demonstrably the case of hedgehog dependent pathways, in particular those dependent upon Shh (Yao et al., 2016). In particular, Shh is active in brain development and also in the maintenance and proliferation of neural stem cells in the adult brain (Carballo et al., 2018; Schwarz el al., 2012). The action of Shh on neurogenesis appears to be regulated by the primary cilia of hippocampal neurons (Breunig et al., 2008). Shh also participates in axon regeneration (see the bibliometric analysis by Chou et al., 2022). The interplay of the Notch and Shh pathways, where Notch signalling potentiates the cell proliferative effect of Shh has been a recurrent finding in the recent years (Yao et al., 2016). Crosstalk between Shh and Wnt has also been documented (Carballo et al., 2018). Dysfunctions in Shh signalling is present in neurodegenerative diseases, metabolic disorders and depression (Yao et al., 2016; Tayyab et al., 2018; Garg et al., 2022).

#### 3.4.5 Notch

Increased Notch signalling has been observed following intermittent fasting and exercise (Brandt et al., 2010; Baik et al., 2020). During development, Notch-dependant pathways ensure neural stem cells maintenance in the developing brain (Ehm et al., 2010). The Notch signaling pathway also plays an important role in the regulation and plasticity of neural stem cells and neural progenitor cells in several niches of the adult brain (Brandt et al., 2010; Ehm et al., 2010; Bagheri-Mohammni, 2022). Disfunctions of Notch signaling are present in AD and other neurodegenerative diseases (Ables et al., 2011). Notch is a master regulator of neuronal life cycle and synaptic plasticity (Ables et al., 2011). “Notch signaling deeply participates in the development and homeostasis of multiple tissues and organs” (Zhou et al., 2022, p. 1). Transcriptional activation is the most known way Notch activity is enhanced, but protein activation is also possible (‘non-canonical’ signalling). (Ables et al., 2011). Precursors of Notch receptors are generated by transcription and translation in the endoplasmic reticulum, after which they undergo maturation in the Golgi apparatus (Zhou et al., 2022). Inhibition of GSK-3 by PI3K-Akt increases the activity of Notch. Notch signalling can enhance the activity of the Hedgehog pathway, including its Ssh ligand (Jacobs & Huang, 2021). The hedgehog pathway also regulates Notch signaling (Jacobs & Huang, 2021). Notch activity can be both carcinogenic and anticarinogenic (Zhou et al., 2022).

#### 3.4.6 Wnt

The Wnt pathways is also important in its neurotrophic activity, including in adult neurogenesis (Arredondo et al., 2020; Alkailani et al., 2022): “[T]he Wnt signaling pathway plays multiple roles in adult hippocampal neurogenesis, including regulation of proliferation, fate-commitment, and maturation of adult-born neurons.” (Arredondo et al., 2022, p. 636). The Wnt/b-catenin pathway is potentiated by AMPK and GSK3β which phosphorylate β-catenin (Zhao et al., 2010; Hermida et al., 2017). Wnt also plays a major role in the differentiation of neuronal precursor cells (Arredondo et al., 2020). General blockade of Wnt almost completely impedes neurogenesis (Arredondo et al., 2020). (Neural) cell proliferation and differentiation which results from autophagy, which is regulated by Wnt (Lorzadeh et al., 2021). Wnt also participates in presynaptic differentiation (e.g., remodeling of the axon and synapse button), presynaptic and post synaptic assembly in the CNS, particularly in the hippocampus, as well as the regulation of the distribution of synapses (Salinas, 2013). Wnt also participates in axon regeneration (Chou et al., 2022). While some have mentioned that deficient Wnt signaling is linked with brain tumor genesis (Alkailani et al., 2022), others have found that increased Wnt signaling due to high glucose might be responsible for the link between diabetes and cancer (García-Giménez et al., 2014). Wnt has been reported to activate mTOR through GSK-3 inhibition, which promotes cell growth (Inoki et al., 2006). The association between Wnt signaling and carcinogenesis thus remains to be clarified. Wnt activity is inhibited by Notch and mTOR (Zeng et al., 2018; Acar et al., 2021). A bibliometric analysis of Wnt is provided by Xu et al. (2021).

#### 3.4.7 Poly(ADP-ribose) polymerases (PARPs)

PARPs participate in anti-aging functions such as the promotion of chromosome integrity, metabolic and cell cycle regulation, inflammation, and protein expression and RNA transcription and processing) (Cantó et al., 2013; Bai, 2015). Epigenetic alterations in PARP activities are linked to neurodegenerative diseases and other aging-related diseases due to genomic instability (Amorim et al., 2022; Mao & Zhang, 2022). EIC are modulator of the activity of PARPs (D’Souza et al., 2017; Hofer et al., 2022). Through ADP-ribosylation (the reversible post-translational modification of proteins), the function of the substrate is altered (Li et al., 2022; Lüscher et al., 2022). ADP-ribosylation plays an important role in neurodevelopment, signal transduction, the maintenance of DNA integrity, RNA expression, as well as (neuron) cell fate under conditions of cellular stress (Li et al., 2022; Lüscher et al., 2022). Li et al. (2022) detail the process of ADP-ribosylation and its functioning in regulating physiological and pathological processes, including the roles of different molecules as writers (e.g., NAD+, PARPs), readers (e.g., PARPs, histones) and erasers (e.g., macrodomain-containing proteins) of ADP-ribosylation.

PARPs play a major role in ADP-ribosylation (Cantó et al., 2013; Lüscher et al., 2022). There are 17 PARPs, which are not detailed here (see Bai, 2015), except for the mention that ADP-ribosylation signaling is mainly regulated by PARP1 and to a lesser extent by other PARPs (Cantó et al., 2013; Lüscher et al., 2022). PARPs are activated following DNA damage (Xie et al., 2020). Indeed, the most important function of PARPs is DNA repair, particularly under cellular stress (Cantó et al., 2013).

They are also involved in the regulation of gene expression, immune cell maturation and influence energy metabolism and response to inflammation (Cantó et al., 2013; Bai, 2015). The chronic activity of PARPs can induce NAD+ depletion, which can result in the shutdown of mitochondrial function and glycolytic slowdown through the reduction of NAD+ availability for other processes such as the activation of SIRTs and elevated PARP activation through ROS increase can lead to several diseases affecting major organs (Cantó et al., 2013; Bai, 2015), including increased tumor growth; therefore PARP inhibitors are actively researched (Li et al., 2022).

## 4 Presentation of the annotated bibliography

In the sub-section focusing on exercise, the following literature reviews are summarized: Radak et al. (2007) focused on the role of exercise in regulating ROS in the brain and discussed the implications with regards to neuroprotection; Powers et al. (2020) described exercise as a producer of ROS and a cause for oxidative stress—mentioning the hormesis hypothesis (the experience of mild stress prepares neurons and tissues to more important stressors); Walsh et al. (2020) which focused on the link between exercise, BDNF, as well as cognition; Reddy et al. (2023) which discussed the cellular processes involved in exercise-induced neuroregulation; Chow et al. (2022) focused exerkines’ health implications, including cognitive decline; Mattson & Arumugam (2018) discussed epigenetic changes leading to mitochondrial disfunctions (e.g., decreased NAD+:NADH ratio, leading to decreased SIRT signaling) and the alleviating role of exercise and IF; Lucassen et al. (2010) focused on the neuroprotective role of exercise and sleep, as well as the deteriorating effects of stress and inflammation.

In the section on IF, the following literature reviews are summarized. Mattson et al. (2018): focused on the importance of intermittent metabolic switching (involved in both IF and CR) in NSPAN and the neurodegenerative danger of lifestyles without opportunities for intermittent metabolic switching; Seidler & Barrow (2022) examined the role of BDNF in the relation of IF with cognitive function; Cherif et al. (2016) examined IF, CR and Ramadan fasting in relation to cognitive function—mentioning the upregulation by IF; Wang et al. (2020) focused on the influence of IF on inflammatory markers—which are related to NSPAN.

In the section on CR, the literature reviews that follow are summarized: Almendariz-Palacios et al. (2020—updated review): focused on the signaling pathways involved in reduced cellular aging stemming from CR (relevant aspects summarized under this sub-section) and CRMs (relevant aspects summarized in the next sub-section); Ntsapi & Loos (2016): focused on the regulation of autophagy by means of different cycle durations of CR, distinguishing between macroautophagy (starting from 30 min of glucose depletion), microautophagy, and CMA (starting from 10 h of glucose depletion)—CMA as being more targeted than the other forms of autophagy; de Mello et al. (2019); focused on the triumvirate insulin, autophagy, and neurodegeneration—examining the signaling pathways involved; Maharajan et al. (2020): discusses the regulation of stem cells aging and stem cell niches by means of CR—with a focus on signaling pathways; Zhang et al. (2021): focused on the optimization of brain extracellular microenvironment through CR and its role towards neuroprotection; Yu H. et al. (2020): examined the restauration and maintenance of cognitive function through CR.

In the section on CRMs, the literature reviews that follow are summarized: Almendariz-Palacios et al. (2020—summary continues); Bonkowski & Sinclair (2016): explained the use of NAD+ precursors and other SIRTs activators in relation with disease prevention—including neurodegeneration and longevity, with a focus on signaling pathways; Isde et al. (2020): focused on the activation of SIRT1 in particular through phytochemicals, CRMs included; Hofer et al. (2021): examined clinical trials focusing on CRMs and discusses a list of CRMs available through nutrition; Moosavi et al. (2016): focused on the neurotrophic actions of polyphenols, discussing NSPAN; Oluwole et al. (2022) detailed the role of different polyphenol (sub)classes: tannins, phenolic acid, lignans, flavonoids and stilbenes in health including the main outcomes of interest; Rendeiro et al. (2015): examined the effect (direct and indirect) of flavonoids in alleviating neurodegeneration in particular; Zhang et al. (2021): focused on the (pre)-clinical uses of resveratrol, addressing neuroprotection through its anti-inflammatory effect; Moradi et al. (2022): focused on the mechanisms of neuronal regeneration following polyphenol intake, addressing ROS scavenging, reduced beta-amyloid accumulation, regulation of apoptosis and gene expression; Grewal et al. (2021): detailed the mechanisms of neuroprotection afforded by quercetin and implications regarding neurodegenerative diseases; Ravula et al. (2021): provided an updated literature review on the prospects for treatment of neurodegenerative disorders offered by fisetin.

In the sub-section on other nutrients inducing neurotrophic effects, summaries of the following reviews are provided. Hebergen (2016): examined the role of n-3 polyunsaturated fatty acids and polyphenols in the promotion of adult neurogenesis; Carneiro et al. (2021): provided an updated systematic literature review on the neuroprotective effects of coffee, focusing on several of its compounds; Chen et al. (2010): addressed the protective role of caffeine on the BBB specifically; Cui et al. (2017): focused on the role of vitamin D in brain development, neuroprotection and immunity, distinguishing between genomic and non-genomic actions.

## 5 Discussion

In the first pages of this contribution (Section A), firstly, the literature on the promotion of NSPAN through EIC and CRMs has been briefly presented. The question of the g-to-k metabolic switch has been addressed and the k-to-g metabolic switching has been mentioned. It was made clear that it is the repetition of these cycles that affords the most health benefits, as “repeating cycles of a metabolic challenge that induces ketosis (fasting and/or exercise) followed by a recovery period (eating, resting and sleeping), may optimize brain function and resilience throughout the lifespan” (Mattson et al. (2018, p. 81). Thus, in section B, glucose metabolism and expenditure as well as oxidative stress have been discussed, the latter being the result of cellular respiration (electron slippage through the, ETC, leading increase in ROS). The introduction continued with the review of the main nucleotides (ATP, ADP, AMP), metabolites (NAD+, NADH, BHB), and neurotrophic factors (primarily BDNF), as well as protein kinases, and signaling proteins involved, such as PI3K, Akt, mTOR, AMPK, GSK3β, ULK, MAPK, PGC-1α, NF-κB, and sirtuins as well as Notch, Shh and Wnt; and selected signaling pathways. While the focus here has been on neurogenesis and neuroprotection, other important functions involved have been discussed (e.g., the regulation of energy metabolism, oxidative stress and inflammation, immune function, apoptosis, autophagy, gene expression and protein synthesis as well as tumor suppression), since beyond their primary role, these functions support the main outcomes of interest. Beckervordersandforth (2017) discuss the role of mitochondrial metabolism in adult neurogenesis. Guo et al. (2022) review the literature on aging in more general terms, related diseases and interventions. Carrasco et al. (2022) and Mittelbrunn & Kroemer (2021) examine the role of T cells in neurodegenerative diseases among others health consequences related to *inflammaging*.

In Section C, a selective list of reviews of interest has been included and a summary of each is provided in the Appendix. The selection of reviews was organized in five sub-sections covering, in relation to neuroprotection and/or neurogenesis: exercise, IF, CR, CRMs as well as other nutrients inducing neurotrophic effects. The aim here was that each sub-section provided overall a general overview of the benefits of its topic (e.g., CR) in relation to neuroprotection and neurogenesis, with each summarized literature review adding specific aspects to this goal.

### 5.1 Other implications for healthy individuals and policy

Pharmacological cognitive enhancement (PCE) has not yet been mentioned *per se* in this contribution. While improving or maintaining NSPAN requires months if not years, PCE usually focuses on the hope of rapid gains and is particularly relied upon by students and pressured professionals (Franke & Lieb, 2013; Mayor et al., 2020). Users of PCE rely upon pharmacological solutions (e.g., methylphenidate, Adderall, or modafinil), generally deviating from the inteded use of these medications, or rely upon illicit drugs. The attitude of the public towards PCE is generally rather negative (Mayor et al., 2019), due to the conception of PCE as focusing on short term performance at school or on the workplace that is considered artificial and therefore unfair and undeserved (Faber et al., 2016). The adoption of NSPAN, with a focus on individual health and collective welfare rather than on achievement might be more acceptable to the public: O’Connor and Joffe (2015) have for instance indicated that individuals are more tolerant of CE when its aim is to compensate for a cognitive deficiency or the maintenance of healthy brain function. This might be even more true in periods of collective challenges, such as the COVID-19 pandemic (Mayor et al., 2022a; Mayor et al., 2022b; Massell et al., 2022), as the importance of the greater good might be paramount.

The regulation of PCE is an important consideration for government policy, which aims to tackle the task of balancing benefits and risks, that are not negligible in the case of PCE as usually conceived; i.e., with a focus on neurotransmitters and the short term functioning of brain regions (Outram & Racine, 2011). Advocating in favor of NSPAN through EIC and CRMs might be a good strategy, as the benefits (e.g., reduced health burden for the individual and the collectivity) far surpass those of PCE—while allowing for maintained or improved cognitive functioning in the long run; and their risks are inappreciable when performed within reason: for instance, exercise is a source of ROS (Bloomer et al., 2005) and should therefore not be performed in excess. Calorie restriction should not lead to malnutrition.

### 5.2 Further studies

The literature presented in the main text, and the reviews summarized in the annotated bibliography have exhibited the implications of EIC and the potential of CRMs towards the enhancement of NSPAN, including in the context of neurodegenerative diseases. Notwithstanding the focus of basic research on rodents, it is believed that the discovered mechanisms are present in humans as well as they relate to the functioning of the eukaryotic cell, which has largely been conserved in mammals (Mattson et al., 2018). But whether or not adult neurogenesis exists in human remains to be definitely established (Denoth-Lippuner & Jessberger, 2021).

In the majority of cases, studies do not compare the effect of the regulation of different signaling pathways on NSPAN, and more often than not, the effect of exercise, calorie restriction, intermittent fasting, and calorie restriction mimetics are not compared either. It thus remains unclear which intervention is more appropriate to achieve improved NSPAN, or in the best case this is a matter of the judgement of the researcher. Further, the same signaling pathway might operate in opposite directions in relation to NSPAN. For instance, the activation of NF-κB has been linked to neuroprotective and neurodegenerative actions (Mincheva-Tasheva et al., 2013; Capece et al., 2022) and it might be related to the decreased risk of depression through the upregulation of BDNF, but NF-κB is also related to increased neuroinflammation, a risk factor for depression (Roy et al., 2023). It is also unclear whether the heightened activation of Wnt is beneficial or not overall, as it is involved in both carcinogenesis and carcinoprevention (Inoki et al., 2006; García-Giménez et al., 2014). Another such example is SIRT6, which is elevated in chronically exercised individuals and performs both protective and pejorative actions in relation to cancer (Yang et al., 2020; Song et al., 2022); recommendations with regards to NSPAN should consider which effect is more important (protective/pejorative) with regards to the different pathways regulated by EIC and CRM in establishing minimal and optimal thresholds across modalities.

Studies should investigate dose-response and robust effect sizes in all these areas and compare them across modalities of EIC/CRM and signaling pathways as a prerequisite to the setting of thresholds for recommendations with regards to EIC and CRM. Such elements are very infrequently reported in literature reviews (but see Lucassen et al., 2010) and are absent from the large majority of studies in the field. Examples of exceptions are Walsch et al. (2020), which mention that 30 min of exercise are necessary for significant changes in BDNF to occur, whereas, in a study in a related field, Moore et al. (2020) note that blood glucose is affected by single short bouts of exercise in a dose-response manner, with benefits starting after only 1 minute. It follows the target duration of exercise or IF depends on the parameter one wishes to increase, but the information on such aspects and the reliability of these estimates are frequently lacking in the literature (Li et al., 2023). Further research could also compare, in relation to dose-response and effect sizes, studies focusing on EIC and CRMs to those on ketogenic diets which show promise in the prevention of AD (e.g., Neth et al., 2020).

The comparison of the effect of EIC and CRMs on different signaling pathways and modalities of EIC/CRM should ideally be performed within the same study as to minimize the risk of differences in effects being due to methodological differences between studies. In a review (Sharman et al., 2019) only few human trials focusing on phytocomponents in the treatment of Alzheimer’s patients were successful. It should also be noted that the sample size should be sufficient to detect small or moderate improvement with adequate power, for instance a sample size of 128 at least for a two-tailed test of the difference in symptoms between two balanced independent groups with a Cohen *D* of 0.5 (medium effect size) and a statistical power of 0.8 (calculated with G*Power 3) at a *p*-value of 0.05. Below the minimal required sample size to achieve a relevant power, most studies will fail to detect the desired effect size. Further, the use of meta-analyses is not well implanted in the field; the examples of Pillon et al., 2020; Wang et al., 2020 were mentioned above. An increased reliance upon meta-analyses could provide precious information on these aspects, should their use not be precluded by the heterogeneity in existing studies or the lack of reporting of effect sizes (Melsen et al., 2014).

An important element should be the determination of minimum and optimal thresholds for the adoption of EIC and CRM modalities in humans (e.g., Rendeiro et al., 2015; Yu Q. et al., 2020; Kelly et al., 2020), if this is possible, or to find reliable and reproducible means for targeting the ideal quantities to individual needs, which is a hallmark of precision medicine (Wang et al., 2023). Importantly, the needs of members of specific groups should be considered.

In the future, research should clarify whether EIC and CRM can play an important role in *targeted* metabolic reprogramming, which is already successfully used in the treatment of cancer, and might be beneficial with regards to NSPAN (El-Sahli & Wang, 2020; Mela et al., 2020; Wang Q. et al., 2022; Pateras et al., 2023). This also entails, ideally, setting objectives in realistic expected improvements prior to running such research programs.

Studies relying upon mass-spectrometry and nuclear magnetic resonance have the potential to further highlight the value of EIC and CRMs in terms of NSPAN and might help in identifying additional signaling metabolites and other effectors affected by EIC and CRMs which could be investigated in further detail in a second step, but mass-spectrometry and nuclear magnetic resonance are uncommonly used for related research questions (Kristal et al., 2007; Luan et al., 2019; Kelly et al., 2020; Baker & Rutter, 2023).

Timing is important in signal transduction, as also within the cell processes unfold through time, with uninterrupted successions of events (Koseska & Bastiaens, 2017). An overwhelming majority of studies examine the effect of EIC and CRMs at a single timepoint, blurring the complexity of the orchestration of events. Live cell imaging has potential in further determining distinctions in signaling dynamics in the cross-talk between different pathways (Valls & Esposito, 2022) in starved cells and those with high availability of nutrients.

## 6 Conclusion

This review and annotated bibliography has synthesized recent efforts at understanding the mechanisms of through which EIC and CRMs afford enhanced NSPAN. The mentioned benefits of the different approaches in terms of the enhancement of NSPAN, as well as explanations of the pathways and mechanisms through which this goal can be achieved, have highlighted the potential of EIC and CRMs for a healthier aging.

## Footnotes

1 E.g.: increased adenosine monophosphate (AMP) to adenosine triphosphate (ATP) ratios sensed by the 5' adenosine monophosphate-activated protein kinase (AMPK) (Hardie, 2018).

## Supplementary material

Supplementary Table S1 The annotated bibliography is presented in the document ‘Table 1.docx’.

## References

[B1] Ables J. L. Breunig J. J. Eisch A. J. Rakic P. (2011). Not (ch) just development: Notch signalling in the adult brain. Nat. Rev. Neurosci. 12, 269–283. 10.1038/nrn3024 21505516 PMC3159580

[B2] Abraham W. C. (2008). Metaplasticity: Tuning synapses and networks for plasticity. Nat. Rev. 9, 387–399. 10.1038/nrn2356 18401345

[B3] Acar A. Hidalgo-Sastre A. Leverentz M. K. Mills C. G. Woodcock S. Baron M. (2021). Inhibition of Wnt signalling by Notch via two distinct mechanisms. Sci. Rep. 11, 9096. 10.1038/s41598-021-88618-5 33907274 PMC8079408

[B4] Albani D. Polito L. Signorini A. Forloni G. (2010). Neuroprotective properties of resveratrol in different neurodegenerative disorders. Biofactors 36, 370–376. 10.1002/biof.118 20848560

[B5] Alessi D. R. Sakamoto K. Bayascas J. R. (2006). LKB1-Dependent signaling pathways. Annu. Rev. Biochem. 75, 137–163. 10.1146/annurev.biochem.75.103004.142702 16756488

[B6] Alkailani M. I. Aittaleb M. Tissir F. (2022). WNT signaling at the intersection between neurogenesis and brain tumorigenesis. Front. Mol. Neurosci. 15, 1017568. 10.3389/fnmol.2022.1017568 36267699 PMC9577257

[B7] Almendariz-Palacios C. Mousseau D. D. Eskiw C. H. Gillespie Z. E. (2020). Still living better through chemistry: An update on caloric restriction and caloric restriction mimetics as tools to promote health and lifespan. Int. J. Mol. Sci. 21, 9220. 10.3390/ijms21239220 33287232 PMC7729921

[B8] Aman Y. Schmauck-Medina T. Hansen M. Morimoto R. I. Simon A. K. Bjedov I. (2021). Autophagy in healthy aging and disease. Nat. Aging 1, 634–650. 10.1038/s43587-021-00098-4 34901876 PMC8659158

[B9] American College of Sports Medicine (2013). ACSM's guidelines for exercise testing and prescription. New Yort: Lippincott Williams & Wilkins. 10.1249/JSR.0b013e31829a68cf 23851406

[B10] Amorim J. A. Coppotelli G. Rolo A. P. Palmeira C. M. Ross J. M. Sinclair D. A. (2022). Mitochondrial and metabolic dysfunction in ageing and age-related diseases. Nat. Rev. Endocrinol. 18, 243–258. 10.1038/s41574-021-00626-7 35145250 PMC9059418

[B11] Anacker C. Hen R. (2017). Adult hippocampal neurogenesis and cognitive flexibility — Linking memory and mood. Nat. Rev. Neurosci. 18, 335–346. 10.1038/nrn.2017.45 28469276 PMC6261347

[B12] Anderson R. Prolla T. (2009). PGC-1alpha in aging and anti-aging interventions. Biochimica Biophysica Acta (BBA)-General Subj. 1790, 1059–1066. 10.1016/j.bbagen.2009.04.005 PMC2743759 19371772

[B13] Aon M. A. Cortassa S. O'rourke B. (2010). Redox-optimized ROS balance: A unifying hypothesis. Biochimica Biophysica Acta (BBA)-Bioenergetics 1797, 865–877. 10.1016/j.bbabio.2010.02.016 PMC2891851 20175987

[B14] Arredondo S. B. Valenzuela-Bezanilla D. Mardones M. D. Varela-Nallar L. (2020). Role of Wnt signaling in adult hippocampal neurogenesis in health and disease. Front. Cell. Dev. Biol. 8, 860. 10.3389/fcell.2020.00860 33042988 PMC7525004

[B15] Arredondo S. B. Valenzuela-Bezanilla D. Santibanez S. H. Varela-Nallar L. (2022). Wnt signaling in the adult hippocampal neurogenic niche. Stem Cells 40, 630–640. 10.1093/stmcls/sxac027 35446432

[B16] Aubrey B. J. Kelly G. L. Janic A. Herold M. J. Strasser A. (2018). How does p53 induce apoptosis and how does this relate to p53-mediated tumour suppression? Cell. Death Differ. 25, 104–113. 10.1038/cdd.2017.169 29149101 PMC5729529

[B17] Autry A. E. Monteggia L. M. (2012). Brain-derived neurotrophic factor and neuropsychiatric disorders. Pharmacol. Rev. 64, 238–258. 10.1124/pr.111.005108 22407616 PMC3310485

[B18] Bagheri-Mohammadi S. (2022). Adult neurogenesis and the molecular signalling pathways in brain: The role of stem cells in adult hippocampal neurogenesis. Int. J. Neurosci. 132, 1165–1177. 10.1080/00207454.2020.1865953 33350876

[B19] Bai P. (2015). Biology of poly(ADP-ribose) polymerases: The factotums of cell maintenance. Mol. Cell. 58, 947–958. 10.1016/j.molcel.2015.01.034 26091343

[B20] Baik S. Rajeev V. Fann D. Y. Jo D. Arumugam T. V. (2020). Intermittent fasting increases adult hippocampal neurogenesis. Brain Behav. 10, e01444. 10.1002/brb3.1444 31804775 PMC6955834

[B21] Baker S. A. Rutter J. (2023). Metabolites as signalling molecules. In Nature reviews molecular cell Biology. Advance online publication. 10.1038/s41580-022-00572-w 36635456

[B22] Balusu S. Praschberger R. Lauwers E. De Strooper B. Verstreken P. (2023). Neurodegeneration cell per cell. Neuron 111, 767–786. 10.1016/j.neuron.2023.01.016 36787752

[B23] Bathina S. Das U. N. (2015). Brain-derived neurotrophic factor and its clinical implications. Archives Med. Sci. 6, 1164–1178. 10.5114/aoms.2015.56342 PMC4697050 26788077

[B24] Baur J. A. Sinclair D. A. (2006). Therapeutic potential of resveratrol: The *in vivo* evidence. Nat. Rev. Drug Discov. 5, 493–506. 10.1038/nrd2060 16732220

[B25] Bayliss J. A. Lemus M. B. Stark R. Santos V. V. Thompson A. Rees D. J. (2016). Ghrelin-AMPK signaling mediates the neuroprotective effects of calorie restriction in Parkinson's disease. J. Neurosci. 36, 3049–3063. 10.1523/jneurosci.4373-15.2016 26961958 PMC4783502

[B26] Beavers K. M. Brinkley T. E. Nicklas B. J. (2010). Effect of exercise training on chronic inflammation. Clin. Chim. Acta 411, 785–793. 10.1016/j.cca.2010.02.069 20188719 PMC3629815

[B27] Beckervordersandforth R. (2017). Mitochondrial metabolism-mediated regulation of adult neurogenesis. Brain Plast. 3, 73–87. 10.3233/BPL-170044 29765861 PMC5928529

[B28] Berdugo-Vega G. Arias-Gil G. López-Fernández A. Artegiani B. Wasielewska J. M. Lee C.-C. (2020). Increasing neurogenesis refines hippocampal activity rejuvenating navigational learning strategies and contextual memory throughout life. Nat. Commun. 11, 135. 10.1038/s41467-019-14026-z 31919362 PMC6952376

[B29] Bhaduri I. Trisal A. Singh A. K. (2023). “Autophagy as a promising therapeutic target in age-associated neurodegenerative disorders,” in Emerging anti-aging strategies. Editor Rizvi S. I. (Singapore: Springer), 135–154. 10.1007/978-981-19-7443-4_3

[B30] Bhoumik S. Yadawa A. K. Srivastava P. Rizvi S. I. (2023). “Intermittent fasting as an anti-aging strategy,” in Emerging anti-aging strategies. Editor Rizvi S. I. (Singapore: Springer), 191–206. 10.1007/978-981-19-7443-4_10

[B31] Blagosklonny M. V. (2019). Fasting and rapamycin: Diabetes versus benevolent glucose intolerance. Cell. Death Dis. 10, 607. 10.1038/s41419-019-1822-8 31406105 PMC6690951

[B32] Bliss T. V. P. Collingridge G. L. Morris R. G. M. Reymann K. G. (2018). Long-term potentiation in the hippocampus: Discovery, mechanisms and function. Neuroforum 24, A103–A120. 10.1515/nf-2017-A059

[B33] Bloom G. S. (2014). Amyloid-β and tau: The trigger and bullet in Alzheimer disease pathogenesis. JAMA neurol. 71, 505–508. 10.1001/jamaneurol.2013.5847 24493463 PMC12908160

[B34] Bloomer R. J. Goldfarb A. H. Wideman L. McKenzie M. J. Consitt L. A. (2005). Effects of acute aerobic and anaerobic exercise on blood markers of oxidative stress. J. Strength & Cond. Res. 19, 276–285. 10.1519/14823.1 15903362

[B35] Bonkowski M. S. Sinclair D. A. (2016). Slowing ageing by design: The rise of NAD+ and sirtuin-activating compounds. Nat. Rev. Mol. Cell. Biol. 17, 679–690. 10.1038/nrm.2016.93 27552971 PMC5107309

[B36] Bortner C. D. Cidlowski J. A. (2020). Ions, the movement of water and the apoptotic volume decrease. Front. Cell. Dev. Biol. 8, 611211. 10.3389/fcell.2020.611211 33324655 PMC7723978

[B37] Brandt M. D. Maass A. Kempermann G. Storch A. (2010). Physical exercise increases Notch activity, proliferation and cell cycle exit of type‐3 progenitor cells in adult hippocampal neurogenesis. Eur. J. Neurosci. 32, 1256–1264. 10.1111/j.1460-9568.2010.07410.x 20950279

[B38] Breunig J. J. Sarkisian M. R. Arellano J. I. Morozov Y. M. Ayoub A. E. Sojitra S. (2008). Primary cilia regulate hippocampal neurogenesis by mediating sonic hedgehog signaling. Proc. Natl. Acad. Sci. 105, 13127–13132. 10.1073/pnas.0804558105 18728187 PMC2529104

[B39] Brieger K. Schiavone S. Miller Jr. Krause K. (2012). Reactive oxygen species: From health to disease. Swiss Med. Wkly. 142, w13659. 10.4414/smw.2012.13659 22903797

[B40] Brunet A. Goodell M. A. Rando T. A. (2022). Ageing and rejuvenation of tissue stem cells and their niches. Nat. Rev. Mol. Cell. Biol. 24, 45–62. 10.1038/s41580-022-00510-w 35859206 PMC9879573

[B41] Budanov A. V. Karin M. (2008). P53 target genes sestrin1 and sestrin2 connect genotoxic stress and mTOR Signaling. Cell. 134, 451–460. 10.1016/j.cell.2008.06.028 18692468 PMC2758522

[B42] Buntwal L. Sassi M. Morgan A. H. Andrews Z. B. Davies J. S. (2019). Ghrelin-mediated hippocampal neurogenesis: Implications for health and disease. Trends Endocrinol. Metabolism 30, 844–859. 10.1016/j.tem.2019.07.001 31445747

[B43] Burtscher J. Romani M. Bernardo G. Popa T. Ziviani E. Hummel F. C. (2022). Boosting mitochondrial health to counteract neurodegeneration. Prog. Neurobiol. 215, 102289. 10.1016/j.pneurobio.2022.102289 35636655

[B44] Cantó C. Gerhart-Hines Z. Feige J. N. Lagouge M. Noriega L. Milne J. C. (2009). AMPK regulates energy expenditure by modulating NAD+ metabolism and SIRT1 activity. Nature 458, 1056–1060. 10.1038/nature07813 19262508 PMC3616311

[B45] Cantó C. Sauve A. A. Bai P. (2013). Crosstalk between poly(ADP-ribose) polymerase and sirtuin enzymes. Mol. Aspects Med. 34, 1168–1201. 10.1016/j.mam.2013.01.004 23357756 PMC3676863

[B46] Cao W. Dou Y. Li A. (2018). Resveratrol boosts cognitive function by targeting SIRT1. Neurochem. Res. 43, 1705–1713. 10.1007/s11064-018-2586-8 29943083

[B47] Capece D. Verzella D. Flati I. Arboretto P. Cornice J. Franzoso G. (2022). NF-κB: Blending metabolism, immunity, and inflammation. Trends Immunol. 43, 757–775. 10.1016/j.it.2022.07.004 35965153

[B48] Carafa V. Rotili D. Forgione M. Cuomo F. Serretiello E. Hailu G. S. (2016). Sirtuin functions and modulation: From chemistry to the clinic. Clin. Epigenetics 8, 61–21. 10.1186/s13148-016-0224-3 27226812 PMC4879741

[B49] Carballo G. B. Honorato J. R. de Lopes G. P. de Sampaio e Spohr T. C. L. (2018). A highlight on Sonic hedgehog pathway. Cell. Commun. Signal. 16, 11–15. 10.1186/s12964-018-0220-7 29558958 PMC5861627

[B50] Cardinaux J. R. Magistretti P. J. Martin J. L. (1997). Brain-derived neurotrophic factor stimulates phosphorylation of stathmin in cortical neurons. Mol. Brain Res. 51, 220–228. 10.1016/S0169-328X(97)00241-6 9427524

[B51] Carneiro S. M. Oliveira M. B. P. Alves R. C. (2021). Neuroprotective properties of coffee: An update. Trends Food Sci. Technol. 113, 167–179. 10.1016/j.tifs.2021.04.052

[B52] Caron E. Ghosh S. Matsuoka Y. Ashton‐Beaucage D. Therrien M. Lemieux S. (2010). A comprehensive map of the mTOR signaling network. Mol. Syst. Biol. 6, 453. 10.1038/msb.2010.108 21179025 PMC3018167

[B53] Carosi J. M. Sargeant T. J. (2019). Rapamycin and Alzheimer disease: A double-edged sword? Autophagy 15, 1460–1462. 10.1080/15548627.2019.1615823 31066320 PMC6613906

[B54] Carracedo A. Pandolfi P. (2008). The PTEN–PI3K pathway: Of feedbacks and cross-talks. Oncogene 27, 5527–5541. 10.1038/onc.2008.247 18794886

[B55] Carrasco E. Gómez de las Heras M. M. Gabandé-Rodríguez E. Desdín-Micó G. Aranda J. F. Mittelbrunn M. (2022). The role of T cells in age-related diseases. Nat. Rev. Immunol. 22, 97–111. 10.1038/s41577-021-00557-4 34099898

[B56] Cavaleri F. Bashar E. (2018). Potential Synergies of *β* -hydroxybutyrate and butyrate on the modulation of metabolism, inflammation, cognition, and general health. J. Nutr. Metabolism, 2018, 7195760. 10.1155/2018/7195760 PMC5902005 29805804

[B57] Chabot F. Caron A. Laplante M. St-Pierre D. H. (2014). Interrelationships between ghrelin, insulin and glucose homeostasis: Physiological relevance. World J. Diabetes 5, 328–341. 10.4239/wjd.v5.i3.328 24936254 PMC4058737

[B58] Chantranupong L. Scaria S. M. Saxton R. A. Gygi M. P. Shen K. Wyant G. A. (2016). The CASTOR proteins are arginine sensors for the mTORC1 pathway. Cell. 165, 153–164. 10.1016/j.cell.2016.02.035 26972053 PMC4808398

[B59] Chatam O. Chapnik N. Froy O. (2022). Resveratrol induces the fasting state and alters circadian metabolism in hepatocytes. Plant Foods Hum. Nutr. 77, 128–134. 10.1007/s11130-022-00954-7 35178649

[B60] Chauvin S. Sobel A. (2015). Neuronal stathmins: A family of phosphoproteins cooperating for neuronal development, plasticity and regeneration. Prog. Neurobiol. 126, 1–18. 10.1016/j.pneurobio.2014.09.002 25449700

[B61] Chen A. Xiong L.-J. Tong Y. Mao M. (2013). Neuroprotective effect of brain-derived neurotrophic factor mediated by autophagy through the PI3K/Akt/mTOR pathway. Mol. Med. Rep. 8, 1011–1016. 10.3892/mmr.2013.1628 23942837

[B62] Chen G. Kroemer G. Kepp O. (2020). Mitophagy: An emerging role in aging and age-associated diseases. Front. Cell. Dev. Biol. 8, 200. 10.3389/fcell.2020.00200 32274386 PMC7113588

[B63] Chen J. Zhou R. Feng Y. Cheng L. (2022a). Molecular mechanisms of exercise contributing to tissue regeneration. Signal Transduct. Target. Ther. 7, 383. 10.1038/s41392-022-01233-2 36446784 PMC9709153

[B64] Chen X. Ghribi O. Geiger J. D. (2010). Caffeine protects against disruptions of the blood-brain barrier in animal models of Alzheimer’s and Parkinson’s diseases. J. Alzheimer’s Dis. 20, S127–S141. 10.3233/JAD-2010-1376 20164568 PMC3086010

[B65] Chen Y. Huang T. Yu Z. Yu Q. Wang Y. Hu J. (2022b). The functions and roles of sestrins in regulating human diseases. Cell. Mol. Biol. Lett. 27, 2. 10.1186/s11658-021-00302-8 34979914 PMC8721191

[B66] Cheng P.-L. Song A.-H. Wong Y.-H. Wang S. Zhang X. Poo M.-M. (2011). Self-amplifying autocrine actions of BDNF in axon development. Proc. Natl. Acad. Sci. 108, 18430–18435. 10.1073/pnas.1115907108 22025720 PMC3215064

[B67] Cherif A. Roelands B. Meeusen R. Chamari K. (2016). Effects of intermittent fasting, caloric restriction, and Ramadan intermittent fasting on cognitive performance at rest and during exercise in adults. Sports Med. 46, 35–47. 10.1007/s40279-015-0408-6 26438184

[B68] Chou Y. Nawabi H. Li J. (2022). Research hotspots and trends for axon regeneration (2000–2021): A bibliometric study and systematic review. Inflamm. Regen. 42, 60–15. 10.1186/s41232-022-00244-4 36476643 PMC9727899

[B69] Chow L. S. Gerszten R. E. Taylor J. M. Pedersen B. K. van Praag H. Trappe S. (2022). Exerkines in health, resilience and disease. Nat. Rev. Endocrinol. 18, 273–289. 10.1038/s41574-022-00641-2 35304603 PMC9554896

[B70] Chu E. Mychasiuk R. Hibbs M. L. Semple B. D. (2021). Dysregulated phosphoinositide 3-kinase signaling in microglia: Shaping chronic neuroinflammation. J. Neuroinflammation 18, 276. 10.1186/s12974-021-02325-6 34838047 PMC8627624

[B71] Chun O. K. Floegel A. Chung S.-J. Chung C. E. Song W. O. Koo S. I. (2010). Estimation of antioxidant intakes from diet and supplements in U.S. Adults. J. Nutr. 140, 317–324. 10.3945/jn.109.114413 20032488

[B72] Cortes C. J. De Miguel Z. (2022). Precision exercise medicine: Sex specific differences in immune and CNS responses to physical activity. Brain Plast. 8, 65–77. 10.3233/bpl-220139 36448044 PMC9661359

[B73] Craske M. G. Stein M. B. Eley T. C. Milad M. R. Holmes A. Rapee R. M. (2017). Anxiety disorders. Nat. Rev. Dis. Prim. 3, 17024. 10.1038/nrdp.2017.24 28470168 PMC11009418

[B74] Cuesto G. Enriquez-Barreto L. Carames C. Cantarero M. Gasull X. Sandi C. (2011). Phosphoinositide-3-kinase activation controls synaptogenesis and spinogenesis in hippocampal neurons. J. Neurosci. 31, 2721–2733. 10.1523/JNEUROSCI.4477-10.2011 21414895 PMC6623769

[B75] Cuesto G. Jordán-Álvarez S. Enriquez-Barreto L. Ferrús A. Morales M. Acebes Á. (2015). GSK3β inhibition promotes synaptogenesis in drosophila and mammalian neurons. PLOS ONE 10, e0118475. 10.1371/journal.pone.0118475 25764078 PMC4357437

[B76] Cui X. Gooch H. Petty A. McGrath J. J. Eyles D. (2017). Vitamin D and the brain: Genomic and non-genomic actions. Mol. Cell. Endocrinol. 453, 131–143. 10.1016/j.mce.2017.05.035 28579120

[B77] Culmsee C. Mattson M. P. (2005). p53 in neuronal apoptosis. Biochem. Biophysical Res. Commun. 331, 761–777. 10.1016/j.bbrc.2005.03.149 15865932

[B78] Davies J. S. (2022). Ghrelin mediated hippocampal neurogenesis. Vitamins Hormones 118, 337–367. 10.1016/bs.vh.2021.12.003 35180932

[B79] de Mello N. P. Orellana A. M. Mazucanti C. H. de Morais Lima G. Scavone C. Kawamoto E. M. (2019). Insulin and autophagy in neurodegeneration. Front. Neurosci., 13, 491. 10.3389/fnins.2019.00491 31231176 PMC6558407

[B80] Demine S. Renard P. Arnould T. (2019). Mitochondrial uncoupling: A key controller of biological processes in physiology and diseases. Cells 8, 795. 10.3390/cells8080795 31366145 PMC6721602

[B81] Denoth-Lippuner A. Jessberger S. (2021). Formation and integration of new neurons in the adult hippocampus. Nat. Rev. Neurosci. 22, 223–236. 10.1038/s41583-021-00433-z 33633402

[B82] Di Meo S. Reed T. T. Venditti P. Victor V. M. (2016). Role of ROS and RNS Sources in physiological and pathological conditions. Oxidative Med. Cell. Longev., 2016, 1245049. 10.1155/2016/1245049 PMC4960346 27478531

[B83] Diano S. Farr S. A. Benoit S. C. McNay E. C. da Silva I. Horvath B. (2006). Ghrelin controls hippocampal spine synapse density and memory performance. Nat. Neurosci. 9, 381–388. 10.1038/nn1656 16491079

[B84] Dolatshahi M. Ranjbar Hameghavandi M. H. Sabahi M. Rostamkhani S. (2021). Nuclear factor‐kappa B (NF‐κB) in pathophysiology of Parkinson disease: Diverse patterns and mechanisms contributing to neurodegeneration. Eur. J. Neurosci. 54, 4101–4123. 10.1111/ejn.15242 33884689

[B85] dos Santos M. G. Schimith L. E. André-Miral C. Muccillo-Baisch A. L. Arbo B. D. Hort M. A. (2022). Neuroprotective effects of resveratrol in *in vivo* and *in vitro* experimental models of Parkinson’s disease: A systematic review. Neurotox. Res., 40, 319–345. 10.1007/s12640-021-00450-x 35013904

[B86] D’Souza R. F. Markworth J. F. Aasen K. M. Zeng N. Cameron-Smith D. Mitchell C. J. (2017). Acute resistance exercise modulates microRNA expression profiles: Combined tissue and circulatory targeted analyses. PLOS ONE 12, e0181594. 10.1371/journal.pone.0181594 28750051 PMC5531502

[B87] Duarte A. I. Santos P. Oliveira C. R. Santos M. S. Rego A. C. (2008). Insulin neuroprotection against oxidative stress is mediated by Akt and GSK-3beta signaling pathways and changes in protein expression. Biochimica Biophysica Acta (BBA) - Mol. Cell. Res. 1783, 994–1002. 10.1016/j.bbamcr.2008.02.016 18348871

[B88] Ehm O. Göritz C. Covic M. Schäffner I. Schwarz T. J. Karaca E. (2010). RBPJkappa-dependent signaling is essential for long-term maintenance of neural stem cells in the adult hippocampus. J. Neurosci. 30, 13794–13807. 10.1523/jneurosci.1567-10.2010 20943920 PMC6633732

[B89] El-Sahli S. Wang L. (2020). Cancer stem cell-associated pathways in the metabolic reprogramming of breast cancer. Int. J. Mol. Sci. 21, 9125. 10.3390/ijms21239125 33266219 PMC7730588

[B90] Elkhwanky M.-S. Hakkola J. (2018). Extranuclear sirtuins and metabolic stress. Antioxidants Redox Signal. 28, 662–676. 10.1089/ars.2017.7270 28707980

[B91] Emili M. Guidi S. Uguagliati B. Giacomini A. Bartesaghi R. Stagni F. (2022). Treatment with the flavonoid 7,8-Dihydroxyflavone: A promising strategy for a constellation of body and brain disorders. Crit. Rev. Food Sci. Nutr. 62, 13–50. 10.1080/10408398.2020.1810625 32914634

[B92] Erbaba B. Arslan-Ergul A. Adams M. M. (2021). Effects of caloric restriction on the antagonistic and integrative hallmarks of aging. Ageing Res. Rev. 66, 101228. 10.1016/j.arr.2020.101228 33246078

[B93] Ersahin T. Tuncbag N. Cetin-Atalay R. (2015). The PI3K/AKT/mTOR interactive pathway. Mol. Biosyst. 11, 1946–1954. 10.1039/C5MB00101C 25924008

[B94] Faber N. S. Savulescu J. Douglas T. (2016). Why is cognitive enhancement deemed unacceptable? The role of fairness, deservingness, and hollow achievements. Front. Psychol. 7, 232. 10.3389/fpsyg.2016.00232 26925027 PMC4759582

[B95] Fagerli E. Escobar I. Ferrier F. J. Jackson C. W. Perez-Lao E. J. Perez-Pinzon M. A. (2022). Sirtuins and cognition: Implications for learning and memory in neurological disorders. Front. Physiology 13, 908689. 10.3389/fphys.2022.908689 PMC9355297 35936890

[B96] Fan J. Dawson T. M. Dawson V. L. (2017). “Cell death mechanisms of neurodegeneration,” in Neurodegenerative diseases: Pathology, mechanisms, and potential therapeutic targets. Editors Beart P. Robinson M. Rattray M. Maragakis N. J., 403–425. 10.1007/978-3-319-57193-5_16

[B97] Fang E. F. Bohr V. A. (2017). NAD^+^: The convergence of DNA repair and mitophagy. Autophagy 13, 442–443. 10.1080/15548627.2016.1257467 27929719 PMC5324847

[B98] Fang Y. Tang S. Li X. (2019). Sirtuins in metabolic and epigenetic regulation of stem cells. Trends Endocrinol. Metabolism 30, 177–188. 10.1016/j.tem.2018.12.002 PMC6382540 30630664

[B99] Fares J. Bou Diab Z. Nabha S. Fares Y. (2019). Neurogenesis in the adult hippocampus: History, regulation, and prospective roles. Int. J. Neurosci. 129, 598–611. 10.1080/00207454.2018.1545771 30433866

[B100] Fayard E. Tintignac L. A. Baudry A. Hemmings B. A. (2005). Protein kinase B/Akt at a glance. J. Cell. Sci. 118, 5675–5678. 10.1242/jcs.02724 16339964

[B101] Fisher-Wellman K. Bloomer R. J. (2009). Acute exercise and oxidative stress: A 30 year history. Dyn. Med. 8, 1. 10.1186/1476-5918-8-1 19144121 PMC2642810

[B102] Foltran R. B. Diaz S. L. (2016). BDNF isoforms: A round trip ticket between neurogenesis and serotonin? J. Neurochem. 138, 204–221. 10.1111/jnc.13658 27167299

[B103] Franke A. G. Lieb K. (2013). in Pharmacological neuroenhancement: Substances and epidemiology. Editors Hildt E. Franke A. G. Enhancement C. (Dordrecht: Springer), 17–27.

[B104] Frankland P. W. Köhler S. Josselyn S. A. (2013). Hippocampal neurogenesis and forgetting. Trends Neurosci. 36, 497–503. 10.1016/j.tins.2013.05.002 23768770

[B105] Gahete M. D. Córdoba-Chacón J. Kineman R. D. Luque R. M. Castaño J. P. (2011). Role of ghrelin system in neuroprotection and cognitive functions: Implications in Alzheimer's disease. Peptides 32, 2225–2228. 10.1016/j.peptides.2011.09.019 21983104 PMC3228413

[B106] García-Jiménez C. García-Martínez J. M. Chocarro-Calvo A. De la Vieja A. (2014). A new link between diabetes and cancer: Enhanced WNT/β-catenin signaling by high glucose. J. Mol. Endocrinol. 52, R51–R66. 10.1530/JME-13-0152 24049067

[B107] García-Rodríguez D. Giménez-Cassina A. (2021). Ketone bodies in the brain beyond fuel metabolism: From excitability to gene expression and cell signaling. Front. Mol. Neurosci. 14, 732120. 10.3389/fnmol.2021.732120 34512261 PMC8429829

[B108] Garg C. Kaur A. Singh T. G. Sharma V. K. Singh S. K. (2022). Therapeutic implications of sonic hedgehog pathway in metabolic disorders: Novel target for effective treatment. Pharmacol. Res. 179, 106194. 10.1016/j.phrs.2022.106194 35364246

[B109] Gillespie Z. E. Pickering J. Eskiw C. H. (2016). Better living through chemistry: Caloric restriction (CR) and CR mimetics alter genome function to promote increased health and lifespan. Front. Genet. 7, 142. 10.3389/fgene.2016.00142 27588026 PMC4988992

[B110] Gomez-Cabrera M. C. Domenech E. Viña J. (2008). Moderate exercise is an antioxidant: Upregulation of antioxidant genes by training. Free Radic. Biol. Med. 44, 126–131. 10.1016/j.freeradbiomed.2007.02.001 18191748

[B111] Gómez-Palacio-Schjetnan A. Escobar M. L. (2013). “Neurotrophins and synaptic plasticity,” in Neurogenesis and neural plasticity. Editors Belzung C. Wigmore P. (Berlin: Springer), 117–136. 10.1007/7854_2012_231 23519767

[B112] Grewal A. K. Singh T. G. Sharma D. Sharma V. Singh M. Rahman Md. H. (2021). Mechanistic insights and perspectives involved in neuroprotective action of quercetin. Biomed. Pharmacother. 140, 111729. 10.1016/j.biopha.2021.111729 34044274

[B113] Guo J. Huang X. Dou L. Yan M. Shen T. Tang W. (2022). Aging and aging-related diseases: From molecular mechanisms to interventions and treatments. Signal Transduct. Target. Ther. 7, 391. 10.1038/s41392-022-01251-0 36522308 PMC9755275

[B114] Haas R. Cucchi D. Smith J. Pucino V. Macdougall C. E. Mauro C. (2016). Intermediates of metabolism: From bystanders to signalling molecules. Trends Biochem. Sci. 41, 460–471. 10.1016/j.tibs.2016.02.003 26935843

[B115] Hahn-Windgassen A. Nogueira V. Chen C.-C. Skeen J. E. Sonenberg N. Hay N. (2005). Akt activates the mammalian Target of Rapamycin by regulating cellular ATP level and AMPK activity. J. Biol. Chem. 280, 32081–32089. 10.1074/jbc.M502876200 16027121

[B116] Handschin C. (2016). Caloric restriction and exercise “mimetics’’: Ready for prime time? Pharmacol. Res. 103, 158–166. 10.1016/j.phrs.2015.11.009 26658171 PMC4970791

[B117] Hardie D. G. (2018). Keeping the home fires burning: AMP-activated protein kinase. J. R. Soc. Interface 15, 20170774. 10.1098/rsif.2017.0774 29343628 PMC5805978

[B118] Hass D. T. Barnstable C. J. (2021). Uncoupling proteins in the mitochondrial defense against oxidative stress. Prog. Retin. Eye Res. 83, 100941. 10.1016/j.preteyeres.2021.100941 33422637 PMC8263805

[B119] He F. Li J. Liu Z. Chuang C.-C. Yang W. Zuo L. (2016). Redox mechanism of reactive oxygen species in exercise. Front. Physiology 7, 486. 10.3389/fphys.2016.00486 PMC5097959 27872595

[B120] He L. Wang J. Yang Y. Li J. Tu H. (2022). Mitochondrial sirtuins in Parkinson’s disease. Neurochem. Res. 47, 1491–1502. 10.1007/s11064-022-03560-w 35220492

[B121] Heberden C. (2016). Modulating adult neurogenesis through dietary interventions. Nutr. Res. Rev. 29, 163–171. 10.1017/S0954422416000081 27364160

[B122] Heger M. (2017). Drug screening: Don't discount all curcumin trial data. Nature 543, 40. 10.1038/543040c PMC6814206 28252078

[B123] Hermida M. A. Kumar J. D. Leslie N. R. (2017). GSK3 and its interactions with the PI3K/AKT/mTOR signalling network. Adv. Biol. Regul. 65, 5–15. 10.1016/j.jbior.2017.06.003 28712664

[B124] Hinchy E. C. Gruszczyk A. V. Willows R. Navaratnam N. Hall A. R. Bates G. (2018). Mitochondria-derived ROS activate AMP-activated protein kinase (AMPK) indirectly. J. Biol. Chem. 293, 17208–17217. 10.1074/jbc.RA118.002579 30232152 PMC6222118

[B125] Hofer S. J. Carmona‐Gutierrez D. Mueller M. I. Madeo F. (2022). The ups and downs of caloric restriction and fasting: From molecular effects to clinical application. EMBO Mol. Med. 14, e14418. 10.15252/emmm.202114418 34779138 PMC8749464

[B126] Hofer S. J. Davinelli S. Bergmann M. Scapagnini G. Madeo F. (2021). Caloric restriction mimetics in nutrition and clinical trials. Front. Nutr. 8, 717343. 10.3389/fnut.2021.717343 34552954 PMC8450594

[B127] Holmström K. M. Finkel T. (2014). Cellular mechanisms and physiological consequences of redox-dependent signalling. Nat. Rev. Mol. Cell. Biol. 15, 411–421. 10.1038/nrm3801 24854789

[B128] Hottiger M. O. (2015). SnapShot: ADP-ribosylation signaling. Mol. Cell. 58, 1134–1134.e1. 10.1016/j.molcel.2015.06.001 26091348

[B129] Hu E. Du H. Zhu X. Wang L. Shang S. Wu X. (2018). Beta-hydroxybutyrate promotes the expression of BDNF in hippocampal neurons under adequate glucose supply. Neuroscience 386, 315–325. 10.1016/j.neuroscience.2018.06.036 29966721

[B130] Huang J. Dibble C. C. Matsuzaki M. Manning B. D. (2008). The TSC1-TSC2 Complex is required for proper activation of mTOR Complex 2. Mol. Cell. Biol. 28, 4104–4115. 10.1128/MCB.00289-08 18411301 PMC2423120

[B131] Igwe O. Sone M. Matveychuk D. Baker G. B. Dursun S. M. (2021). A review of effects of calorie restriction and fasting with potential relevance to depression. Prog. Neuro-Psychopharmacology Biol. Psychiatry 111, 110206. 10.1016/j.pnpbp.2020.110206 33316333

[B132] Inoki K. Ouyang H. Zhu T. Lindvall C. Wang Y. Zhang X. (2006). TSC2 Integrates Wnt and energy signals via a Coordinated phosphorylation by AMPK and GSK3 to regulate cell growth. Cell. 126, 955–968. 10.1016/j.cell.2006.06.055 16959574

[B133] Ishizuka Y. Kakiya N. Witters L. A. Oshiro N. Shirao T. Nawa H. (2013). AMP‐activated protein kinase counteracts brain‐derived neurotrophic factor‐induced mammalian target of rapamycin complex 1 signaling in neurons. J. Neurochem. 127, 66–77. 10.1111/jnc.12362 23841933

[B134] Iside C. Scafuro M. Nebbioso A. Altucci L. (2020). SIRT1 activation by natural phytochemicals: An overview. Front. Pharmacol. 11, 1225. 10.3389/fphar.2020.01225 32848804 PMC7426493

[B135] Jacobs C. T. Huang P. (2021). Complex crosstalk of Notch and Hedgehog signalling during the development of the central nervous system. Cell. Mol. Life Sci. 78, 635–644. 10.1007/s00018-020-03627-3 32880661 PMC11072263

[B136] Jaworski T. Banach-Kasper E. Gralec K. (2019). GSK-3 *β* at the intersection of neuronal plasticity and neurodegeneration. Neural Plast. 2019, 4209475. 10.1155/2019/4209475 31191636 PMC6525914

[B137] Jeanneteau F. Deinhardt K. Miyoshi G. Bennett A. M. Chao M. V. (2010). The MAP kinase phosphatase MKP-1 regulates BDNF-induced axon branching. Nat. Neurosci. 13, 1373–1379. 10.1038/nn.2655 20935641 PMC2971689

[B138] Jiao F. Gong Z. (2020). The beneficial roles of SIRT1 in neuroinflammation-related diseases. Oxidative Med. Cell. Longev., 2020, 6782872. 10.1155/2020/6782872 PMC7519200 33014276

[B139] Johnstone A. Mobley W. (2020). Local TrkB signaling: Themes in development and neural plasticity. Cell. Tissue Res. 382, 101–111. 10.1007/s00441-020-03278-7 32936344

[B140] Kaeberlein M. (2010). Resveratrol and rapamycin: Are they anti‐aging drugs? Bioessays 32, 96–99. 10.1002/bies.200900171 20091754

[B141] Kallies G. Rapp M. A. Fydrich T. Fehm L. Tschorn M. Terán C. (2019). Serum brain-derived neurotrophic factor (BDNF) at rest and after acute aerobic exercise in major depressive disorder. Psychoneuroendocrinology 102, 212–215. 10.1016/j.psyneuen.2018.12.015 30583245

[B142] Karabiyik C. Vicinanza M. Son S. M. Rubinsztein D. C. (2021). Glucose starvation induces autophagy via ULK1-mediated activation of PIKfyve in an AMPK-dependent manner. Dev. Cell. 56, 1961–1975.e5. 10.1016/j.devcel.2021.05.010 34107300

[B143] Kelly R. S. Kelly M. P. Kelly P. (2020). Metabolomics, physical activity, exercise and health: A review of the current evidence. Biochimica Biophysica Acta (BBA)-Molecular Basis Dis. 1866, 165936. 10.1016/j.bbadis.2020.165936 PMC7680392 32827647

[B144] Kempermann G. Gage F. H. Aigner L. Song H. Curtis M. A. Thuret S. (2018). Human adult neurogenesis: Evidence and remaining questions. Cell. Stem Cell. 23, 25–30. 10.1016/j.stem.2018.04.004 29681514 PMC6035081

[B145] Kim D. H. Park M. H. Ha S. Bang E. J. Lee Y. Lee A. K. (2019). Anti-inflammatory action of β-hydroxybutyrate via modulation of PGC-1α and FoxO1, mimicking calorie restriction. Aging 11, 1283–1304. 10.18632/aging.101838 30811347 PMC6402511

[B146] Kökten T. Hansmannel F. Ndiaye N. C. Heba A. C. Quilliot D. Dreumont N. (2021). Calorie restriction as a new treatment of inflammatory diseases. Adv. Nutr. 12, 1558–1570. 10.1093/advances/nmaa179 33554240 PMC8321869

[B147] Kolb H. Kempf K. Röhling M. Lenzen-Schulte M. Schloot N. C. Martin S. (2021). Ketone bodies: From enemy to friend and guardian angel. BMC Med. 19, 313–315. 10.1186/s12916-021-02185-0 34879839 PMC8656040

[B148] Koppel S. J. Swerdlow R. H. (2018). Neuroketotherapeutics: A modern review of a century-old therapy. Neurochem. Int. 117, 114–125. 10.1016/j.neuint.2017.05.019 28579059 PMC5711637

[B149] Korla K. Mitra C. K. (2014). Modelling the Krebs cycle and oxidative phosphorylation. J. Biomol. Struct. Dyn. 32, 242–256. 10.1080/07391102.2012.762723 23528175

[B150] Koseska A. Bastiaens P. I. (2017). Cell signaling as a cognitive process. EMBO J. 36, 568–582. 10.15252/embj.201695383 28137748 PMC5331751

[B151] Kowiański P. Lietzau G. Czuba E. Waśkow M. Steliga A. Moryś J. (2018). Bdnf: A key factor with multipotent impact on brain signaling and synaptic plasticity. Cell. Mol. Neurobiol. 38, 579–593. 10.1007/s10571-017-0510-4 28623429 PMC5835061

[B152] Krieglstein K. Strelau J. Schober A. Sullivan A. Unsicker K. (2002). TGF- β and the regulation of neuron survival and death. J. Physiology 96, 25–30. 10.1016/S0928-4257(01)00077-8 11755780

[B153] Kristal B. S. Shurubor Y. I. Kaddurah-Daouk R. Matson W. R. (2007). “Metabolomics in the study of aging and caloric restriction,” in Biological aging: Methods and Protocols. Editor Tollefsbol T. O. (Totowa: Humana Press), 393–409. 10.1007/978-1-59745-361-5_25 17634593

[B154] Kumar M. Bansal N. (2022). Implications of phosphoinositide 3-kinase-Akt (PI3K-Akt) pathway in the pathogenesis of Alzheimer’s disease. Mol. Neurobiol. 59, 354–385. 10.1007/s12035-021-02611-7 34699027

[B155] Laberge R.-M. Adler D. DeMaria M. Mechtouf N. Teachenor R. Cardin G. B. (2013). Mitochondrial DNA damage induces apoptosis in senescent cells. Cell. Death Dis. 4, e727. 10.1038/cddis.2013.199 23868060 PMC3730395

[B156] Lautrup S. Sinclair D. A. Mattson M. P. Fang E. F. (2019). NAD+ in brain aging and neurodegenerative disorders. Cell. Metab. 30, 630–655. 10.1016/j.cmet.2019.09.001 31577933 PMC6787556

[B157] Leckie R. L. Oberlin L. E. Voss M. W. Prakash R. S. Szabo-Reed A. Chaddock-Heyman L. (2014). BDNF mediates improvements in executive function following a 1-year exercise intervention. Front. Hum. Neurosci. 8, 985. 10.3389/fnhum.2014.00985 25566019 PMC4263078

[B158] Leite J. A. Ghirotto B. Targhetta V. P. Lima J. Câmara N. O. S. (2022). Sirtuins as pharmacological targets in neurodegenerative and neuropsychiatric disorders. Br. J. Pharmacol. 179, 1496–1511. 10.1111/bph.15570 34029375

[B159] Li P. Lei Y. Qi J. Liu W. Yao K. (2022). Functional roles of ADP-ribosylation writers, readers and erasers. Front. Cell. Dev. Biol. 10, 941356. 10.3389/fcell.2022.941356 36035988 PMC9404506

[B160] Li Z. Huang L. Luo Y. Yu B. Tian G. (2023). Effects and possible mechanisms of intermittent fasting on health and disease: A narrative review. Nutr. Rev., nuad026. 10.1093/nutrit/nuad026 36940184

[B161] Liu G. Y. Sabatini D. M. (2020). MTOR at the nexus of nutrition, growth, ageing and disease. Nat. Rev. Mol. Cell. Biol. 21, 183–203. 10.1038/s41580-019-0199-y 31937935 PMC7102936

[B162] Liu T. Zhang L. Joo D. Sun S.-C. (2017). NF-κB signaling in inflammation. Signal Transduct. Target. Ther. 2, 17023. 10.1038/sigtrans.2017.23 29158945 PMC5661633

[B163] Lorzadeh S. Kohan L. Ghavami S. Azarpira N. (2021). Autophagy and the Wnt signaling pathway: A focus on Wnt/β-catenin signaling. Biochimica Biophysica Acta (BBA)-Molecular Cell. Res. 1868, 118926. 10.1016/j.bbamcr.2020.118926 33316295

[B164] Lu Y. Tao F. Zhou M. T. Tang K. F. (2019). The signaling pathways that mediate the anti-cancer effects of caloric restriction. Pharmacol. Res. 141, 512–520. 10.1016/j.phrs.2019.01.021 30641278

[B165] Luan H. Wang X. Cai Z. (2019). Mass spectrometry‐based metabolomics: Targeting the crosstalk between gut microbiota and brain in neurodegenerative disorders. Mass Spectrom. Rev. 38, 22–33. 10.1002/mas.21553 29130504

[B166] Lucassen P. J. Fitzsimons C. P. Salta E. Maletic-Savatic M. (2020). Adult neurogenesis, human after all (again): Classic, optimized, and future approaches. Behav. Brain Res. 381, 112458. 10.1016/j.bbr.2019.112458 31899214

[B167] Lucassen P. J. Meerlo P. Naylor A. S. van Dam A. M. Dayer A. G. Fuchs E. (2010). Regulation of adult neurogenesis by stress, sleep disruption, exercise and inflammation: Implications for depression and antidepressant action. Eur. Neuropsychopharmacol. 20, 1–17. 10.1016/j.euroneuro.2009.08.003 19748235

[B168] Lüscher B. Ahel I. Altmeyer M. Ashworth A. Bai P. Chang P. (2022). ADP‐ribosyltransferases, an update on function and nomenclature. FEBS J. 289, 7399–7410. 10.1111/febs.16142 34323016 PMC9027952

[B169] Madeo F. Carmona-Gutierrez D. Hofer S. J. Kroemer G. (2019). Caloric restriction mimetics against age-associated disease: Targets, mechanisms, and therapeutic potential. Cell. Metab. 29, 592–610. 10.1016/j.cmet.2019.01.018 30840912

[B170] Magee J. C. Grienberger C. (2020). Synaptic plasticity forms and functions. Annu. Rev. Neurosci. 43, 95–117. 10.1146/annurev-neuro-090919-022842 32075520

[B171] Magliulo L. Bondi D. Pini N. Marramiero L. Di Filippo E. S. (2022). The wonder exerkines—novel insights: A critical state-of-the-art review. Mol. Cell. Biochem. 477, 105–113. 10.1007/s11010-021-04264-5 34554363 PMC8755664

[B172] Maharajan N. Vijayakumar K. Jang C. H. Cho G.-W. (2020). Caloric restriction maintains stem cells through niche and regulates stem cell aging. J. Mol. Med. 98, 25–37. 10.1007/s00109-019-01846-1 31713638

[B173] Mao K. Zhang G. (2022). The role of PARP1 in neurodegenerative diseases and aging. FEBS J. 289, 2013–2024. 10.1111/febs.15716 33460497

[B174] Marosi K. Mattson M. P. (2014). BDNF mediates adaptive brain and body responses to energetic challenges. Trends Endocrinol. Metabolism 25, 89–98. 10.1016/j.tem.2013.10.006 PMC3915771 24361004

[B175] Martel J. Chang S. H. Wu C. Y. Peng H. H. Hwang T. L. Ko Y. F. (2021). Recent advances in the field of caloric restriction mimetics and anti-aging molecules. Ageing Res. Rev. 66, 101240. 10.1016/j.arr.2020.101240 33347992

[B176] Martínez-Reyes I. Chandel N. S. (2020). Mitochondrial TCA cycle metabolites control physiology and disease. Nat. Commun. 11, 102. 10.1038/s41467-019-13668-3 31900386 PMC6941980

[B177] Massell J. Lieb R. Meyer A. Mayor E. (2022). Fluctuations of psychological states on Twitter before and during COVID-19. PLOS ONE 17, e0278018. 10.1371/journal.pone.0278018 36516149 PMC9750014

[B178] Mattson M. P. (2019). An evolutionary perspective on why food overconsumption impairs cognition. Trends Cognitive Sci. 23, 200–212. 10.1016/j.tics.2019.01.003 PMC6412136 30670325

[B179] Mattson M. P. Arumugam T. V. (2018). Hallmarks of brain aging: Adaptive and pathological modification by metabolic states. Cell. Metab. 27, 1176–1199. 10.1016/j.cmet.2018.05.011 29874566 PMC6039826

[B180] Mattson M. P. Moehl K. Ghena N. Schmaedick M. Cheng A. (2018). Intermittent metabolic switching, neuroplasticity and brain health. *Nat. Rev.* Neurosci. 19, 63–80. 10.1038/nrn.2017.156 PMC5913738 29321682

[B181] Mayor E. Bietti L. M. Canales-Rodríguez E. J. (2022). Text as signal. A tutorial with case studies focusing on social media (Twitter). In Behavior research methods. Advance online publication. 10.3758/s13428-022-01917-1 PMC9311346 35879505

[B182] Mayor E. Daehne M. Bianchi R. (2019). How perceived substance characteristics affect ethical judgement towards cognitive enhancement. PLOS ONE 14, e0213619. 10.1371/journal.pone.0213619 30870469 PMC6417673

[B183] Mayor E. Daehne M. Bianchi R. (2020). The Dark Triad of personality and attitudes toward cognitive enhancement. BMC Psychol. 8, 119. 10.1186/s40359-020-00486-2 33160397 PMC7648998

[B184] Mayor E. Miché M. Lieb R. (2022). Associations between emotions expressed in internet news and subsequent emotional content on Twitter. Heliyon 8, e12133. 10.1016/j.heliyon.2022.e12133 36561692 PMC9763764

[B185] Mazat J.-P. Devin A. Ransac S. (2020). Modelling mitochondrial ROS production by the respiratory chain. Cell. Mol. Life Sci. 77, 455–465. 10.1007/s00018-019-03381-1 31748915 PMC11104992

[B186] McAllister A. K. Katz L. C. Lo D. C. (1999). Neurotrophins and synaptic plasticity. Annu. Rev. Neurosci. 22, 295–318. 10.1146/annurev.neuro.22.1.295 10202541

[B187] McCubrey J. A. Rakus D. Gizak A. Steelman L. S. Abrams S. L. Lertpiriyapong K. (2016). Effects of mutations in Wnt/β-catenin, hedgehog, Notch and PI3K pathways on GSK-3 activity—diverse effects on cell growth, metabolism and cancer. Biochimica Biophysica Acta (BBA)-Molecular Cell. Res. 1863, 2942–2976. 10.1016/j.bbamcr.2016.09.004 27612668

[B188] McGregor M. M. Nelson A. B. (2019). Circuit mechanisms of Parkinson’s disease. Neuron 101, 1042–1056. 10.1016/j.neuron.2019.03.004 30897356

[B189] Mehmel M. Jovanović N. Spitz U. (2020). Nicotinamide riboside — The current state of research and therapeutic uses. Nutrients 12, 1616. 10.3390/nu12061616 32486488 PMC7352172

[B190] Mela V. Mota B. C. Milner M. McGinley A. Mills K. H. Kelly Á. M. (2020). Exercise-induced re-programming of age-related metabolic changes in microglia is accompanied by a reduction in senescent cells. Brain, Behav. Immun. 87, 413–428. 10.1016/j.bbi.2020.01.012 31978523

[B191] Melsen W. G. Bootsma M. C. J. Rovers M. M. Bonten M. J. M. (2014). The effects of clinical and statistical heterogeneity on the predictive values of results from meta-analyses. Clin. Microbiol. Infect. 20, 123–129. 10.1111/1469-0691.12494 24320992

[B192] Miller R. A. Harrison D. E. Astle C. M. Baur J. A. Boyd A. R. De Cabo R. (2011). Rapamycin, but not resveratrol or simvastatin, extends life span of genetically heterogeneous mice. Journals Gerontology Ser. A 66, 191–201. 10.1093/gerona/glq178 PMC3021372 20974732

[B193] Mincheva-Tasheva S. Soler R. M. (2013). NF-κB signaling pathways: Role in nervous system physiology and pathology. Neurosci. 19, 175–194. 10.1177/1073858412444007 22785105

[B194] Mittelbrunn M. Kroemer G. (2021). Hallmarks of T cell aging. Nat. Immunol. 22, 687–698. 10.1038/s41590-021-00927-z 33986548

[B195] Moore J. Salmons H. Vinoskey C. Kressler J. (2020). A single one-minute, comfortable paced, stair-climbing bout reduces postprandial glucose following a mixed meal. Nutr. Metabolism Cardiovasc. Dis. 30, 1967–1972. 10.1016/j.numecd.2020.06.020 32811738

[B196] Moosavi F. Hosseini R. Saso L. Firuzi O. (2016). Modulation of neurotrophic signaling pathways by polyphenols. Drug Des. Dev. Ther. 10, 23–42. 10.2147/DDDT.S96936 PMC4694682 26730179

[B197] Moradi S. Z. Jalili F. Farhadian N. Joshi T. Wang M. Zou L. (2022). Polyphenols and neurodegenerative diseases: Focus on neuronal regeneration. Crit. Rev. Food Sci. Nutr. 62, 3421–3436. 10.1080/10408398.2020.1865870 33393375

[B198] Morris B. J. (2013). Seven sirtuins for seven deadly diseases of aging. Free Radic. Biol. Med. 56, 133–171. 10.1016/j.freeradbiomed.2012.10.525 23104101

[B199] Murphy M. P. (2009). How mitochondria produce reactive oxygen species. Biochem. J. 417, 1–13. 10.1042/BJ20081386 19061483 PMC2605959

[B200] Nadeeshani H. Li J. Ying T. Zhang B. Lu J. (2022). Nicotinamide mononucleotide (NMN) as an anti-aging health product–promises and safety concerns. J. Adv. Res. 37, 267–278. 10.1016/j.jare.2021.08.003 35499054 PMC9039735

[B201] Navas L. E. Carnero A. (2021). NAD+ metabolism, stemness, the immune response, and cancer. Signal Transduct. Target. Ther. 6, 2. 10.1038/s41392-020-00354-w 33384409 PMC7775471

[B202] Negredo P. N. Yeo R. W. Brunet A. (2020). Aging and rejuvenation of neural stem cells and their niches. Cell. Stem Cell. 27, 202–223. 10.1016/j.stem.2020.07.002 32726579 PMC7415725

[B203] Neth B. J. Mintz A. Whitlow C. Jung Y. Sai K. S. Register T. C. (2020). Modified ketogenic diet is associated with improved cerebrospinal fluid biomarker profile, cerebral perfusion, and cerebral ketone body uptake in older adults at risk for Alzheimer’s disease: A pilot study. Neurobiol. aging 86, 54–63. 10.1016/j.neurobiolaging.2019.09.015 31757576 PMC7266642

[B204] Newman J. C. Verdin E. (2017). β-Hydroxybutyrate: A signaling metabolite. Annu. Rev. Nutr. 37, 51–76. 10.1146/annurev-nutr-071816-064916 28826372 PMC6640868

[B205] Nie T. Yang S. Ma H. Zhang L. Lu F. Tao K. (2016). Regulation of ER stress-induced autophagy by GSK3β-TIP60-ULK1 pathway. Cell. Death Dis. 7, e2563. 10.1038/cddis.2016.423 28032867 PMC5260977

[B206] Niwa-Kawakita M. Ferhi O. Soilihi H. Le Bras M. Lallemand-Breitenbach V. de Thé H. (2017). PML is a ROS sensor activating p53 upon oxidative stress. J. Exp. Med. 214, 3197–3206. 10.1084/jem.20160301 28931625 PMC5679165

[B207] Nordvall G. Forsell P. Sandin J. (2022). Neurotrophin-targeted therapeutics: A gateway to cognition and more? Drug Discov. Today 27, 103318. 10.1016/j.drudis.2022.07.003 35850433

[B208] Ntsapi C. Loos B. (2016). Caloric restriction and the precision-control of autophagy: A strategy for delaying neurodegenerative disease progression. Exp. Gerontol. 83, 97–111. 10.1016/j.exger.2016.07.014 27473756

[B209] O’Connor C. Joffe H. (2015). How the public engages with brain optimization: The media-mind relationship. Sci. Technol. Hum. Values 40, 712–743. 10.1177/0162243915576374 PMC4531115 26336326

[B210] Oluwole O. Fernando W. B. Lumanlan J. Ademuyiwa O. Jayasena V. (2022). Role of phenolic acid, tannins, stilbenes, lignans and flavonoids in human health – A review. Int. J. Food Sci. Technol. 57, 6326–6335. 10.1111/ijfs.15936

[B211] Othman Z. Halim A. S. A. Azman K. F. Ahmad A. H. Zakaria R. Sirajudeen K. N. S. (2022). Profiling the research landscape on cognitive aging: A bibliometric analysis and network visualization. Front. Aging Neurosci. 14, 876159. 10.3389/fnagi.2022.876159 35572132 PMC9093595

[B212] Outram S. M. Racine E. (2011). Examining reports and policies on cognitive enhancement: Approaches, rationale, and recommendations. Account. Res. 18, 323–341. 10.1080/08989621.2011.606734 21916740

[B213] Overall R. W. Walker T. L. Fischer T. J. Brandt M. D. Kempermann G. (2016). Different mechanisms must be considered to explain the increase in hippocampal neural precursor cell proliferation by physical activity. Front. Neurosci. 10, 362. 10.3389/fnins.2016.00362 27536215 PMC4971098

[B214] Pateras I. S. Williams C. Gianniou D. D. Margetis A. T. Avgeris M. Rousakis P. (2023). Short term starvation potentiates the efficacy of chemotherapy in triple negative breast cancer via metabolic reprogramming. J. Transl. Med. 21, 169. 10.1186/s12967-023-03935-9 36869333 PMC9983166

[B215] Peyssonnaux C. Eychène A. (2001). The Raf/MEK/ERK pathway: New concepts of activation. Biol. Cell. 93, 53–62. 10.1016/S0248-4900(01)01125-X 11730323

[B216] Pillon N. J. Gabriel B. M. Dollet L. Smith J. A. Sardón Puig L. Botella J. (2020). Transcriptomic profiling of skeletal muscle adaptations to exercise and inactivity. Nat. Commun. 11, 470. 10.1038/s41467-019-13869-w 31980607 PMC6981202

[B217] Powers S. K. Deminice R. Ozdemir M. Yoshihara T. Bomkamp M. P. Hyatt H. (2020). Exercise-induced oxidative stress: Friend or foe? J. Sport Health Sci. 9, 415–425. 10.1016/j.jshs.2020.04.001 32380253 PMC7498668

[B218] Rabinovitch R. C. Samborska B. Faubert B. Ma E. H. Gravel S.-P. AndrzejewskiRaissi T. C. (2017). AMPK maintains cellular metabolic homeostasis through regulation of mitochondrial reactive oxygen species. Cell. Rep. 21, 1–9. 10.1016/j.celrep.2017.09.026 28978464

[B219] Radak Z. Kumagai S. Taylor A. W. Naito H. Goto S. (2007). Effects of exercise on brain function: Role of free radicals. Appl. Physiology, Nutr. Metabolism 32, 942–946. 10.1139/H07-081 18059620

[B220] Radak Z. Suzuki K. Posa A. Petrovszky Z. Koltai E. Boldogh I. (2020). The systemic role of SIRT1 in exercise mediated adaptation. Redox Biol. 35, 101467. 10.1016/j.redox.2020.101467 32086007 PMC7284913

[B221] Rahman M. Muhammad S. Khan M. A. Chen H. Ridder D. A. Müller-Fielitz H. (2014). The β-hydroxybutyrate receptor HCA2 activates a neuroprotective subset of macrophages. Nat. Commun. 5, 3944. 10.1038/ncomms4944 24845831

[B222] Rai S. N. Dilnashin H. Birla H. Singh S. S. Zahra W. Rathore A. S. (2019). The Role of PI3K/Akt and ERK in neurodegenerative disorders. Neurotox. Res. 35, 775–795. 10.1007/s12640-019-0003-y 30707354

[B223] Ravula A. R. Teegala S. B. Kalakotla S. Pasangulapati J. P. Perumal V. Boyina H. K. (2021). Fisetin, potential flavonoid with multifarious targets for treating neurological disorders: An updated review. Eur. J. Pharmacol. 910, 174492. 10.1016/j.ejphar.2021.174492 34516952

[B224] Reddy I. Yadav Y. Dey C. S. (2023). Cellular and molecular regulation of exercise — A neuronal perspective. Cell. Mol. Neurobiol. 43, 1551–1571. 10.1007/s10571-022-01272-x 35986789 PMC11412429

[B225] Rege S. D. Geetha T. Griffin G. D. Broderick T. L. Babu J. R. (2014). Neuroprotective effects of resveratrol in Alzheimer disease pathology. Front. Aging Neurosci. 6, 218. 10.3389/fnagi.2014.00218 25309423 PMC4161050

[B226] Rendeiro C. Rhodes J. S. Spencer J. P. (2015). The mechanisms of action of flavonoids in the brain: Direct versus indirect effects. Neurochem. Int. 89, 126–139. 10.1016/j.neuint.2015.08.002 26260546

[B227] Richter E. A. Hargreaves M. (2013). Exercise, GLUT4, and skeletal muscle glucose uptake. Physiol. Rev. 93, 993–1017. 10.1152/physrev.00038.2012 23899560

[B228] Richter E. A. Ruderman N. B. (2009). AMPK and the biochemistry of exercise: Implications for human health and disease. Biochem. J. 418, 261–275. 10.1042/BJ20082055 19196246 PMC2779044

[B229] Rius-Pérez S. Torres-Cuevas I. Millán I. Ortega Á. L. Pérez S. (2020). PGC-1*α*, inflammation, and oxidative stress: An integrative view in metabolism. Oxidative Med. Cell. Longev., 2020, 1452696. 10.1155/2020/1452696 PMC7085407 32215168

[B230] Roy S. Ansari M. A. Choudhary K. Singh S. (2023). NLRP3 inflammasome in depression: A review. Int. Immunopharmacol. 117, 109916. 10.1016/j.intimp.2023.109916 36827927

[B231] Sadria M. Layton A. T. (2021). Interactions among mTORC, AMPK and SIRT: A computational model for cell energy balance and metabolism. Cell. Commun. Signal. 19, 57–17. 10.1186/s12964-021-00706-1 34016143 PMC8135154

[B232] Salinas P. C. (2013). “Wnt signaling,” in Cellular migration and formation of neuronal connections. Editors Rubenstein J. L. R. Rakic P. (Elsevier), 623–638. 10.1016/B978-0-12-397266-8.00106-X

[B233] Santo E. E. Paik J. (2018)., 127. Elsevier, 105–118. 10.1016/bs.ctdb.2017.10.002 FOXO in neural cells and diseases of the nervous system Curr. Top. Dev. Biol. 29433734 PMC5881381

[B234] Schofield J. H. Schafer Z. T. (2021). Mitochondrial reactive oxygen species and mitophagy: A complex and nuanced relationship. Antioxidants Redox Signal. 34, 517–530. 10.1089/ars.2020.8058 32079408

[B235] Schwarz T. J. Ebert B. Lie D. C. (2012). Stem cell maintenance in the adult mammalian hippocampus: A matter of signal integration? Dev. Neurobiol. 72, 1006–1015. 10.1002/dneu.22026 22488809

[B236] Seidler K. Barrow M. (2022). Intermittent fasting and cognitive performance – targeting BDNF as potential strategy to optimise brain health. Front. Neuroendocrinol. 65, 100971. 10.1016/j.yfrne.2021.100971 34929259

[B237] Shackelford D. B. Shaw R. J. (2009). The LKB1–AMPK pathway: Metabolism and growth control in tumour suppression. Nat. Rev. Cancer 9, 563–575. 10.1038/nrc2676 19629071 PMC2756045

[B238] Sharman M. J. Verdile G. Kirubakaran S. Parenti C. Singh A. Watt G. (2019). Targeting inflammatory pathways in Alzheimer’s disease: A focus on natural products and phytomedicines. CNS Drugs 33, 457–480. 10.1007/s40263-019-00619-1 30900203

[B239] Shaw R. J. (2009). LKB1 and AMP-activated protein kinase control of mTOR signalling and growth. Acta Physiol. 196, 65–80. 10.1111/j.1748-1716.2009.01972.x PMC2760308 19245654

[B240] Shilovsky G. A. Putyatina T. S. Morgunova G. V. Seliverstov A. V. Ashapkin V. V. Sorokina E. V. (2021). A crosstalk between the biorhythms and gatekeepers of longevity: Dual role of glycogen synthase kinase-3. Biochem. Mosc. 86, 433–448. 10.1134/S0006297921040052 PMC8033555 33941065

[B241] Shimazu T. Hirschey M. D. Newman J. He W. Shirakawa K. Le Moan N. (2013). Suppression of oxidative stress by β-hydroxybutyrate, an endogenous histone deacetylase inhibitor. Science 339, 211–214. 10.1126/science.1227166 23223453 PMC3735349

[B242] Simpson I. A. Appel N. M. Hokari M. Oki J. Holman G. D. Maher F. (1999). Blood-brain barrier glucose transporter: Effects of hypo‐and hyperglycemia revisited. J. Neurochem. 72, 238–247. 10.1046/j.1471-4159.1999.0720238.x 9886075

[B243] Singh A. Kukreti R. Saso L. Kukreti S. (2019). Oxidative stress: A key modulator in neurodegenerative diseases. Molecules 24, 1583. 10.3390/molecules24081583 31013638 PMC6514564

[B244] Song M. Y. Han C. Y. Moon Y. J. Lee J. H. Bae E. J. Park B. H. (2022). Sirt6 reprograms myofibers to oxidative type through CREB-dependent Sox6 suppression. Nat. Commun. 13, 1808. 10.1038/s41467-022-29472-5 35379817 PMC8980083

[B245] Spalding K. L. Bergmann O. Alkass K. Bernard S. Salehpour M. Huttner H. B. (2013). Dynamics of hippocampal neurogenesis in adult humans. Cell. 153, 1219–1227. 10.1016/j.cell.2013.05.002 23746839 PMC4394608

[B246] Spasić M. R. Callaerts P. Norga K. K. (2009). AMP-Activated Protein Kinase (AMPK) molecular crossroad for metabolic control and survival of neurons. Neurosci. 15, 309–316. 10.1177/1073858408327805 19359670

[B247] Sutkowy P. Woźniak A. Mila-Kierzenkowska C. Szewczyk-Golec K. Wesołowski R. Pawłowska M. (2021). Physical activity vs. redox balance in the brain: Brain health, aging and diseases. Antioxidants 11, 95. 10.3390/antiox11010095 35052600 PMC8773223

[B248] Szwed A. Kim E. Jacinto E. (2021). Regulation and metabolic functions of mTORC1 and mTORC2. Physiol. Rev. 101, 1371–1426. 10.1152/physrev.00026.2020 33599151 PMC8424549

[B249] Tang B. L. (2016). Sirt1 and the mitochondria. Mol. Cells 39, 87–95. 10.14348/molcells.2016.2318 26831453 PMC4757807

[B250] Taylor S. S. Kornev A. P. (2011). Protein kinases: Evolution of dynamic regulatory proteins. Trends Biochem. Sci. 36, 65–77. 10.1016/j.tibs.2010.09.006 20971646 PMC3084033

[B251] Tayyab M. Shahi M. H. Farheen S. Mariyath M. P. Khanam N. Castresana J. S. (2018). Sonic hedgehog, Wnt, and brain‐derived neurotrophic factor cell signaling pathway crosstalk: Potential therapy for depression. J. Neurosci. Res. 96, 53–62. 10.1002/jnr.24104 28631844

[B252] Testa G. Biasi F. Poli G. Chiarpotto E. (2014). Calorie restriction and dietary restriction mimetics: A strategy for improving healthy aging and longevity. Curr. Pharm. Des. 20, 2950–2977. 10.2174/13816128113196660699 24079773

[B253] Thorens B. Mueckler M. (2010). Glucose transporters in the 21st century. Am. J. Physiology-Endocrinology Metabolism 298, E141–E145. 10.1152/ajpendo.00712.2009 PMC2822486 20009031

[B254] Tinsley G. M. La Bounty P. M. (2015). Effects of intermittent fasting on body composition and clinical health markers in humans. Nutr. Rev. 73, 661–674. 10.1093/nutrit/nuv041 26374764

[B255] Tuncdemir S. N. Lacefield C. O. Hen R. (2019). Contributions of adult neurogenesis to dentate gyrus network activity and computations. Behav. Brain Res. 374, 112112. 10.1016/j.bbr.2019.112112 31377252 PMC6724741

[B256] Vadlakonda L. Dash A. Pasupuleti M. Anil Kumar K. Reddanna P. (2013). The paradox of Akt-mTOR interactions. Front. Oncol. 3, 165. 10.3389/fonc.2013.00165 23802099 PMC3687210

[B257] Valls P. O. Esposito A. (2022). Signalling dynamics, cell decisions, and homeostatic control in health and disease. Curr. Opin. Cell. Biol. 75, 102066. 10.1016/j.ceb.2022.01.011 35245783 PMC9097822

[B258] van Haeringen M. Milaneschi Y. Lamers F. Penninx B. W. Jansen R. (2022). Dissection of depression heterogeneity using proteomic clusters. Psychol. Med., 1–9. 10.1017/S0033291721004888 PMC10235664 35039097

[B259] Van Praag H. Fleshner M. Schwartz M. W. Mattson M. P. (2014). Exercise, energy intake, glucose homeostasis, and the brain. J. Neurosci. 34, 15139–15149. 10.1523/JNEUROSCI.2814-14.2014 25392482 PMC4228123

[B260] van Praag H. (2008). Neurogenesis and exercise: Past and future directions. NeuroMolecular Med. 10, 128–140. 10.1007/s12017-008-8028-z 18286389

[B261] Vanhaesebroeck B. Stephens L. Hawkins P. (2012). PI3K signalling: The path to discovery and understanding. Nat. Rev. Mol. Cell. Biol. 13, 195–203. 10.1038/nrm3290 22358332

[B262] Vella L. Caldow M. K. Larsen A. E. Tassoni D. Della Gatta P. A. Gran P. (2012). Resistance exercise increases NF-κB activity in human skeletal muscle. Am. J. Physiology-Regulatory, Integr. Comp. Physiology 302, R667–R673. 10.1152/ajpregu.00336.2011 22189669

[B263] Vernier M. Giguère V. (2021). Aging, senescence and mitochondria: The PGC-1/ERR axis. Endocrinology 1, 661–R14. 10.1530/JME-20-0196 33112791

[B264] Vints W. A. J. Levin O. Fujiyama H. Verbunt J. Masiulis N. (2022). Exerkines and long-term synaptic potentiation: Mechanisms of exercise-induced neuroplasticity. Front. Neuroendocrinol. 66, 100993. 10.1016/j.yfrne.2022.100993 35283168

[B265] Walsh E. I. Smith L. Northey J. Rattray B. Cherbuin N. (2020). Towards an understanding of the physical activity-BDNF-cognition triumvirate: A review of associations and dosage. Ageing Res. Rev. 60, 101044. 10.1016/j.arr.2020.101044 32171785

[B266] Wancket L. M. Frazier W. J. Liu Y. (2012). Mitogen-activated protein kinase phosphatase (MKP)-1 in immunology, physiology, and disease. Life Sci. 90, 237–248. 10.1016/j.lfs.2011.11.017 22197448 PMC3465723

[B267] Wang K. Liu H. Hu Q. Wang L. Liu J. Zheng Z. (2022a). Epigenetic regulation of aging: Implications for interventions of aging and diseases. Signal Transduct. Target. Ther. 7, 374. 10.1038/s41392-022-01211-8 36336680 PMC9637765

[B268] Wang Q. Lu M. Zhu X. Gu X. Zhang T. Xia C. (2022b). The role of microglia immunometabolism in neurodegeneration: Focus on molecular determinants and metabolic intermediates of metabolic reprogramming. Biomed. Pharmacother. 153, 113412. 10.1016/j.biopha.2022.113412 36076537

[B269] Wang X. Yang Q. Liao Q. Li M. Zhang P. Santos H. O. (2020). Effects of intermittent fasting diets on plasma concentrations of inflammatory biomarkers: A systematic review and meta-analysis of randomized controlled trials. Nutrition 79, 110974. 10.1016/j.nut.2020.110974 32947129

[B270] Wang Y. Li Z. Mo F. Chen-Mayfield T. J. Saini A. LaMere A. M. (2023). Chemically engineering cells for precision medicine. Chem. Soc. Rev. 52, 1068–1102. 10.1039/D2CS00142J 36633324

[B271] Watson K. Baar K. (2014). mTOR and the health benefits of exercise. Seminars Cell. & Dev. Biol. 36, 130–139. 10.1016/j.semcdb.2014.08.013 25218794

[B272] Woods A. Johnstone S. R. Dickerson K. Leiper F. C. Fryer L. G. D. Neumann D. (2003). LKB1 Is the upstream kinase in the AMP-activated protein kinase cascade. Curr. Biol. 13, 2004–2008. 10.1016/j.cub.2003.10.031 14614828

[B273] Xie N. Zhang L. Gao W. Huang C. Huber P. E. Zhou X. (2020). NAD+ metabolism: Pathophysiologic mechanisms and therapeutic potential. Signal Transduct. Target. Ther. 5, 227. 10.1038/s41392-020-00311-7 33028824 PMC7539288

[B274] Xu J. Q. Tang N. Zhang L. F. Tan C. Su Y. George D. M. (2021). A bibliometric analysis of Wnt signaling pathway: From the top-100 cited articles to emerging trends. Ann. Transl. Med. 9, 1065. 10.21037/atm-21-174 34422977 PMC8339812

[B275] Xue F. Li X. Qin L. Liu X. Li C. Adhikari B. (2021). Anti-aging properties of phytoconstituents and phyto-nanoemulsions and their application in managing aging-related diseases. Adv. Drug Deliv. Rev. 176, 113886. 10.1016/j.addr.2021.113886 34314783

[B276] Yadav D. Tripathi Y. B. Singh P. Kesharwani R. K. Keservani R. K. (2017). “Roles of AMP, ADP, ATP, and AMPK in healthy energy boosting and prolonged life span,” in Sustained energy for enhanced human functions and activity. Editor Bagchi D. (Elsevier), 31–51. 10.1016/B978-0-12-805413-0.00002-8

[B277] Yamamoto H. Schoonjans K. Auwerx J. (2007). Sirtuin functions in health and disease. Mol. Endocrinol. 21, 1745–1755. 10.1210/me.2007-0079 17456799

[B278] Yang J. Li Y. Zhang Y. Fang X. Chen N. Zhou X. (2020). Sirt6 promotes tumorigenesis and drug resistance of diffuse large B-cell lymphoma by mediating PI3K/Akt signaling. J. Exp. Clin. Cancer Res. 39, 142. 10.1186/s13046-020-01623-w 32711549 PMC7382040

[B279] Yao P. J. Petralia R. S. Mattson M. P. (2016). Sonic hedgehog signaling and hippocampal neuroplasticity. Trends Neurosci. 39, 840–850. 10.1016/j.tins.2016.10.001 27865563 PMC5148655

[B280] Yu H. Lin L. Zhang Z. Zhang H. Hu H. (2020b). Targeting NF-κB pathway for the therapy of diseases: Mechanism and clinical study. Signal Transduct. Target. Ther. 5, 209–223. 10.1038/s41392-020-00312-6 32958760 PMC7506548

[B281] Yu M. Zhang H. Wang B. Zhang Y. Zheng X. Shao B. (2021). Key signaling pathways in aging and potential interventions for healthy aging. Cells 10, 660. 10.3390/cells10030660 33809718 PMC8002281

[B282] Yu Q. Zou L. Kong Z. Yang L. (2020a). Cognitive impact of calorie restriction: A narrative review. J. Am. Med. Dir. Assoc. 21, 1394–1401. 10.1016/j.jamda.2020.05.047 32693996

[B283] Zeng H. Lu B. Zamponi R. Yang Z. Wetzel K. Loureiro J. (2018). mTORC1 signaling suppresses Wnt/β-catenin signaling through DVL-dependent regulation of Wnt receptor FZD level. Proc. Natl. Acad. Sci. 115, E10362-E10369–E10369. 10.1073/pnas.1808575115 30297426 PMC6217415

[B284] Zhang L.-X. Li C.-X. Kakar M. U. Khan M. S. Wu P.-F. Amir R. M. (2021b). Resveratrol (RV): A pharmacological review and call for further research. Biomed. Pharmacother. 143, 112164. 10.1016/j.biopha.2021.112164 34649335

[B285] Zhang L. Xu H. Ding N. Li X. Chen X. Chen Z. (2021a). Beneficial effects on brain micro-environment by caloric restriction in alleviating neurodegenerative diseases and brain aging. Front. Physiolology 12, 715443. 10.3389/fphys.2021.715443 PMC8660583 34899367

[B286] Zhao C. Deng W. Gage F. H. (2008). Mechanisms and functional implications of adult neurogenesis. Cell. 132, 645–660. 10.1016/j.cell.2008.01.033 18295581

[B287] Zhao J. Yue W. Zhu M. J. Sreejayan N. Du M. (2010). AMP-activated protein kinase (AMPK) cross-talks with canonical Wnt signaling via phosphorylation of β-catenin at Ser 552. Biochem. Biophysical Res. Commun. 395, 146–151. 10.1016/j.bbrc.2010.03.161 PMC2864303 20361929

[B288] Zhao R. Jiang S. Zhang L. Yu Z. (2019). Mitochondrial electron transport chain, ROS generation and uncoupling (Review). Int. J. Mol. Med. 44, 3–15. 10.3892/ijmm.2019.4188 31115493 PMC6559295

[B289] Zhao Y. Jia M. Chen W. Liu Z. (2022). The neuroprotective effects of intermittent fasting on brain aging and neurodegenerative diseases via regulating mitochondrial function. Free Radic. Biol. Med. 182, 206–218. 10.1016/j.freeradbiomed.2022.02.021 35218914

[B290] Zhou B. Lin W. Long Y. Yang Y. Zhang H. Wu K. (2022). Notch signaling pathway: Architecture, disease, and therapeutics. Signal Transduct. Target. Ther. 7, 95. 10.1038/s41392-022-00934-y 35332121 PMC8948217

[B291] Zia A. Pourbagher-Shahri A. M. Farkhondeh T. Samarghandian S. (2021). Molecular and cellular pathways contributing to brain aging. Behav. Brain Funct. 17, 6–30. 10.1186/s12993-021-00179-9 34118939 PMC8199306

