# Supplementary material for: Neurotrophic effects of intermittent fasting, calorie restriction and exercise: a review and annotated bibliography
Source: Front Aging. 2023 Jun 2;4:1161814. doi: 10.3389/fragi.2023.1161814 (PMC10273285; doi:10.3389/fragi.2023.1161814)
Supplement: Supplementary file 1 [file Table1.DOCX]

Supplementary information for

Neurotrophic Effects of Intermittent Fasting, Calorie Restriction and Exercise: A Review and Annotated Bibliography

Eric Mayor, University of Basel, Switzerland

ericmarcel.mayor@unibas.ch

Note: For acronyms not defined in this document, see the main text.

**Appendix. Annotated bibliography**

**Exercise**

Radak, Z., Kumagai, S., Taylor, A. W., Naito, H., & Goto, S. (2007). Effects of exercise on brain function: Role of free radicals. *Applied Physiology, Nutrition, and Metabolism*, *32*, 942–946. https://doi.org/10.1139/h07-081

The role of exercise in the management of ROS is discussed by Radak et al. (2007), with focus on the implications on the CNS. It is showed that oxidative damage of ROS is ultimately linked to neurodegenerative diseases. The protective role of exercise with a focus on neurotrophins and trophic factors are discussed; and among the neurotrophins, some details are provided on BDNF, for which the up-regulation is related to exercise. Through the signaling pathways it can activate, BDNF has a protective role with regards to ROS notably. CREB is mentioned as a downstream effector of BDNF which is linked to an increase in BDNF dependent upon the level of ROS. Hence there is a feedback loop, which is potentiated by exercise.

Powers, S. K., Deminice, R., Ozdemir, M., Yoshihara, T., Bomkamp, M. P., & Hyatt, H. (2020). Exercise-induced oxidative stress: Friend or foe? *Journal of Sport and Health Science*, *9*(5), 415–425. https://doi.org/10.1016/j.jshs.2020.04.001

Powers et al. (2020) focus on exercise as a producer of ROS and a cause of oxidative stress, in the skeletal muscle primarily. The concept of oxidative stress is defined as an imbalance in the pro-oxidant/antioxidant ratio that leads to cellular damage. A short explanation on free radicals is provided. And several molecule that compose the ROS and reactive nitrogen species (NOS) labels are briefly discussed. Enzymatic and nonenzymatic antioxidants are presented as central in the cellular control of ROS. The history of research on exercise-induced oxidative stress and on the production of ROS produced in skeletal muscles during exercise is presented as well as the impact of ROS on muscle performance. Mention is made of the hormesis theory - exercise is presented as a mild stressor that prepares the body to future challenges.

Walsh, E. I., Smith, L., Northey, J., Rattray, B., & Cherbuin, N. (2020). Towards an understanding of the physical activity-BDNF-cognition triumvirate: A review of associations and dosage. *Ageing Research Reviews, 60*, 101044.

Walsh et al. (2020) focus on the exercise-circulating BDNF-cognition dose-response associations. Physical activity is the most important factors increasing BDNF, of which the neurotrophic functions are mentioned. BDNF and its measurement and concentration are discussed across the life span: 20-30 nanograms per liter with a decrease of 0.5 – 5% per year. Modulators of BDNF, notably, physical activity are presented. Brain aging is then discussed with the mention of mechanisms first, and next the presentation of aging as a result of the presented mechanisms and cognitive decline as a consequence of the latter. The discussion the continues with the involvement of BDNF in aging processes, focusing on the brain and changes in cognitive performance. The association of BDNF with exercise, with mentions a few values of interest are then mentioned. Notably, more than 30 minutes of exercise is necessary to increase serum BDNF and exercise intensity might only be marginally related to the magnitude of the increase in BDNF. Chronic effects of interventions targeting basal BDNF, meta-analyses notably show small effects of exercise on serum BDNF. The next presented elements are the inconclusive link between serum BDNF concentration and cerebral blood flow; that cellular signaling is an important confound in the assessment of the effect of physical activity on BDNF, the lack of research on the acute BDNF response and cognition. Finally, the mode of physical activity, exercise intensity and its duration, as well as work and rest periods, prior training and the BDNF response to exercise in the context of aging are discussed.

Reddy, I., Yadav, Y., & Dey, C. S. (2023). Cellular and molecular regulation of exercise—A neuronal perspective. *Cellular and Molecular Neurobiology*, *43,* 1551–1571 https://doi.org/10.1007/s10571-022-01272-x

Reddy et al. (2023) provide a literature review of the processes involved in exercise-induces neuronal regulation. Research on the physiological response to exercise is synthesized with a focus on the proteins and metabolites, such as exerkines (Irisin, Cathepsin B, interleukin-6 - IL-6, Adiponectin, Osteocacin, Leptin, Fibroblast growth factor-21 - FGF-21), growth factors and signaling proteins, that are regulated by exercise, notably. Irisin in involved in neuroprotection, cognitive improvements and the amelioration of Alzheimer's disease. Cathepsin B is involved in cognitive improvements. IL-6 regulates brain metabolism, notably, and reduces brain inflammation. Adiponectin is involved in dendritic arborization, spinogenesis and neurogenesis notably. Isteocalcin regulates cognition, neuroplasticity, memory and learning. FGF-21 reduces neuroinflammation and improves synaptic plasticity. And leptin is involved in neurogenesis, axonal growth and improved spatial memory notably. Other signaling proteins are mentioned: Orexin A, Musclin, Secreted protein acidic and rich in cysteine (SPARC), Meteorin-like protein (METRNL), BDNF, AMPK, SIRT1, PGC-1*α*, and IGF-1. We have mentioned BDNF, AMPK, SIRT1, PGC-1*α* and IGF-1 in the main text. Orexin A and SPARC have been related to neurodegenerative diseases. Increased musclin is detrimental to metabolism. Musclin secretion is down-regulated by exercise. METRNL is involved in different functions. Finally, exercise mimetics are mentioned.

Chow, L. S., Gerszten, R. E., Taylor, J. M., Pedersen, B. K., van Praag, H., Trappe, S., Febbraio, M. A., Galis, Z. S., Gao, Y., Haus, J. M., Lanza, I. R., Lavie, C. J., Lee, C.-H., Lucia, A., Moro, C., Pandey, A., Robbins, J. M., Stanford, K. I., Thackray, A. E., … Snyder, M. P. (2022). Exerkines in health, resilience and disease. *Nature Reviews Endocrinology*, *18*(5), 273–289. https://doi.org/10.1038/s41574-022-00641-2

In Chow et al. (2022), the health implications of exerkines – substances increased by exercise which are involved in signaling – are presented. Mention is made of the health benefits of exercise are linked to the release of exerkines. Although primarily studied in the skeletal muscle, exerkines can be released by a variety of organs. They might promote health notably by diminishing obesity, reducing risks of cardiaovascular disease and cancer, and protecting from cognitive decline. A list of exerkines is provided (e.g., Adiponectin, BDNF, FGF21, IL-6, Lactate, Musclin, Nitric oxide) and mention of the location of their origin is made (e.g., muscle, brain, brown adipose tissue). The most common measures of exerkines, their advantages and disadvantages are discussed. Exerkines play different roles in the cardiometabolic system, such as the improvement of angiogenesis for Angiopoietin I, the optimization of glucose and lipids for adiponectin) in the adipose tissue, the skeletal muscle, bones, the liver and gut, the nervous, immune and endocrine systems. The muscle-brain crosstalk is also discussed in the review, with mention of the role of myokines (exerkines released by the skeletal muscle) in hippocampal neurogenesis in particular. The distinction between acute and chronic exercise is important in that matter, as is kynurenine, a liver-secreted exerkine which can protect the brain from stress.

Mattson, M. P., & Arumugam, T. V. (2018). Hallmarks of brain aging: adaptive and pathological modification by metabolic states. *Cell Metabolism*, *27*, 1176–1199. https://doi.org/10.1016/j.cmet.2018.05.011

Mattson & Arumugam (2018) focus on exercise and IF in relation to healthy brain aging. Cognitive decline is presented in the context of aging. It accelerates from 50 years of age and increases odds for neurodegenerative disorders. The physiological age of the brain can be modulated by environmental factors, CR, CRMs, and IF notably. Early to mid-life environmental factors can affect the risk of future neurodegenerative diseases. Traumatic brain injury, and the history of intermittent metabolic switching (e.g., through CR, or IF; beneficial) and chronic positive energy balance (detrimental) are the presented, which is followed by mitochondrial dysfunction (notably on axons and dendrites) in the brain aging process. Mitochondrial dysfunction can result notably in decreased NAD+:NADH ratio, which affects signaling (e.g., SIRT). Different issues are caused by mitochondrial dysfunction (e.g., deficient ETC, increased ROS), which can lead to neurodegenerative disorders and focus on CR as an alleviative process. The processes through which oxidative imbalance can lead to impaired brain health as well as the impaired degradation of damaged proteins are discussed. Next, the discussion continues with dysregulated calcium ion homeostasis in neurons (modulator of neuron function and neuronal networks) and deficient cellular stress response as well as the relevant processes and signaling pathways involved. Dysfunctional neuronal activity is the next topic addressed is the review, followed by impaired DNA repair in aging; then, age-related heightened inflammation and impaired neurogenesis, as well as the limited role of telomere attrition in neurons and cell senescence. A discussion of dysregulation in energy metabolism, with a focus on IRS-1, BHB, and omega-3 fatty acids is presented next. Neurodegenerative disorders are related to aging, and the examples of Alzheimer disease and Parkinson’s notably are discussed. Intermittent metabolic challenges and pharmacological interventions are mentioned as means of delaying brain aging, improve cognitive function and neuroprotection. The review then focuses on the processes involved, starting with the depletion of the liver glycogen store and lipolysis and the pathways at play. Examples of pharmacological strategies are provided, for instance ketone esters to improve cognition, nicotinamide riboside to extend lifespan and diminish neurodegeneration, as well as mechanisms of action.

Lucassen, P. J., Meerlo, P., Naylor, A. S., van Dam, A. M., Dayer, A. G., Fuchs, E., Oomen, C. A., & Czéh, B. (2010). Regulation of adult neurogenesis by stress, sleep disruption, exercise and inflammation: Implications for depression and antidepressant action. *European Neuropsychopharmacology*, *20*, 1–17. https://doi.org/10.1016/j.euroneuro.2009.08.003

Lucassen et al. (2010) focus on the implications of exercise, sleep and antidepressants, as well as the deteriorating effects of stress and inflammation with regards to neurogenesis. The importance of stress, as an alarm system, for the mobilization of the organism and the deleterious consequences of chronic and uncontrollable stress and severe acute stress are presented. These manifestations of stress can lead to diverse pathologies, notably major depressive disorder, of which the authors mention the hippocampal alterations. Stress affects functions of the hippocampus and its structure (e.g., volume loss; aberrations in synaptic organization). Adult neurogenesis is then discussed, including the areas where it has been established to occur in humans – and points of controversy; and the different steps of this process. How exercise plays an important role in improved neurogenesis is discussed, including the regulation of adult hippocampal neurogenesis by exercise and how it is affected by stress (mentioning the suppressive effect of the latter). Mechanisms for the detrimental effect of stress in relation to neurogenesis are detailed, with mention of concentration of neurotransmitters and neurotrophic factors. Different aspects relative to perinatal stress are presented, as well as sleep disruption as a stressor with ramifications in impaired neurogenesis - but sleep disruption during one single night seems to be unrelated to the process. Hormones and neurotransmitters affected by sleep disruption are presented. Inflammation as a cell stressor is discussed as well as its importance in hypothalamic–pituitary–adrenocortical axis dysregulation, and the consequences regarding adult neurogenesis. Stress also affects the prefrontal cortex (PFC), cytogenesis in this area in particular: Dysregulation of the activity of the PFC can result from impaired cytogenesis. The neurogenic theory is described. This theory links increased risks of depression with impaired neurogenesis following stress exposure. The conclusion addresses reduced long lasting reduced neurogenesis as a cause for depression, and the protective role of voluntary exercise and antidepressants.

**Intermittent fasting (IF)**

Mattson, M. P., Moehl, K., Ghena, N., Schmaedick, M., & Cheng, A. (2018). Intermittent metabolic switching, neuroplasticity and brain health. *Nature Reviews Neuroscience*, *19*, 81–94. https://doi.org/10.1038/nrn.2017.156

An evolutionary account of the contribution of repeated metabolic switching to improved neuroplasticity is provided by Mattson et al. (2018) in the form of a comprehensive review. IF is presented as the most frequent repeated metabolic switching paradigm, of which different forms are mentioned (alternate day feeding, daily time-restricted feeding) together with exercise. Intermittent metabolic switching involves the glucose-to-ketone (leading to cellular stress resistance) switch and the ketone-to-glucose (leading to cell growth and plasticity). Organisms evolved to perform well under conditions of scarce resources and that metabolic switching is one way to achieve such result, because of the production of ketone bodies (due to lipolysis), which can be used as cellular fuel when glucose is scarce. The neuronal dynamics induced by repeated metabolic switching are discussed, with mention of the cognitive benefits of exercise and neuroprotection as well as synaptic plasticity. This is followed by the signaling pathways elicited by metabolic switching, focusing on the one hand on neurotransmitters and neurotrophic factors, and on the other hand on the management of cellular stress and mitochondrial biogenesis. Following this, the role of hunger hormone ghrelin is introduced, as well as the neurotrophic factors Insulin-like growth factor I (IGF-1) and fibroblast growth factor 2 (FGF-2) as well as BHB. Finally, repeated metabolic switching is presented as a brain health intervention.

Seidler, K., & Barrow, M. (2022). Intermittent fasting and cognitive performance – Targeting BDNF as potential strategy to optimise brain health. *Frontiers in Neuroendocrinology*, *65*, 100971. https://doi.org/10.1016/j.yfrne.2021.100971

The link between IF and cognition is the focus of Seidler & Barrow (2022). As an introduction, how high caloric diets participates in the development of neurodegenerative diseases is discussed. The role of BDNF as a central molecule in healthy aging and adaptation is presented next. The review links CR and IF to BDNF signaling, with mention of its relevance in relation to neurogenesis, synaptic plasticity, long term potentiation, memory and learning, notably. Then the central role of diminished synaptic plasticity and neurogenesis in neurodegeneration is described. In the systematic review part, research on animals is presented, followed by humans. The findings of the systematic review demonstrate that nutritional challenges fulfill adaptative functions, which involve the BDNF/TrkB signaling pathway; and that short vs long term benefits in cognition can take place depending on the duration of BDNF stimulation.

Cherif, A., Roelands, B., Meeusen, R., & Chamari, K. (2016). Effects of intermittent fasting, caloric restriction, and Ramadan intermittent fasting on cognitive performance at rest and during exercise in adults. *Sports Medicine, 46*, 35-47. https://doi.org/10.1007/s40279-015-0408-6

The influence of IF, Ramadan fasting and CR on adult cognitive performance during exercise or at rest is the focus of Cherif et al. (2016). The health benefits of exercise and how it affects the brain, the importance of balanced energy intake and the distinction between calories restriction and IF are mentioned, which is followed by a brief review of the literature on fasting and cognitive function as well as the importance of nutrition for athletic performance. Next CR is presented, with mention of caloric deficit values and benefits associated with CR (e.g., increased lifespan) with accompanying experimental evidence, as which mechanisms for the benefits of CR on cognitive function are presented. The IF is distinguished from religious fasting and alternate day fasting. The cognitive benefits of IF, and the beneficial changes in neurochemistry and neuronal networks that stem from it, including BDNF concentration are discussed. Mention is made of the increase in parasympathetic activity that exercise promotes and the benefits of metabolic switching. Next, the discussion of Ramadan fasting defines its scope and describe its effects (positive and negative) on cognition and other areas (e.g., sleep). A general overview the effects of the diets of interest on cognitive function is proposed, starting with the importance of the relationship between immunity and cognitive function (focusing on cytokines) which is related to interferences of inflammation with in the expression of BDNF and BDNF signaling. After this, the effects of the diets on glucose and lipid metabolism is related to cognition (poorer with hypoglycemia). The importance of lactate (the by-product of glycolysis) for long term memory and of metabolic changes associated with the diets in relation to neuroprotection is discussed. This is followed by the detrimental effects of dehydration on cognition - which start with 1% loss in body weight and the review of evidence relating the combination of exercise and the diets to the healthy brain.

Wang, X., Yang, Q., Liao, Q., Li, M., Zhang, P., Santos, H. O., ... & Abshirini, M. (2020). Effects of intermittent fasting diets on plasma concentrations of inflammatory biomarkers: a systematic review and meta-analysis of randomized controlled trials. *Nutrition, 79*, 110974. doi: 10.1016/j.nut.2020.110974

A meta-analysis focusing on IF and inflammatory biomarkers is proposed by Wang et al. (2020). It is introduced with the mention of the importance of a balanced energy intake in longevity and the risks associated with an over consumption of calories in related to metabolic disorders. The aging processes can be attenuated by IF and energy-restricted diets (variations are discussed). Next energy restricted diets and IF are presented in relation to the activation of AMPK - a central process in the benefits of these diets. The meta-analysis (*k* = 18, 15 articles). examines the effects of the diets on the concentration of C-reactive protein (CRP), tumor necrosis factor - α (TNF- α) and interleukin-6 (IL-6). Findings show the diets compared with ad libitum feeding affected CRP (IF more than energy restricted diets), but not TNF- α nor IL-6. No evidence of publication bias is reported.

**Calorie restriction (CR)**

Almendariz-Palacios, C., Mousseau, D. D., Eskiw, C. H., & Gillespie, Z. E. (2020). Still living better through chemistry: An update on caloric restriction and caloric restriction mimetics as tools to promote health and lifespan. *International Journal of Molecular Sciences, 21,* 9220. doi:10.3390/ijms21239220

An updated review of the literature (see Gillespie et al., 2016 for the original review) on healthy lifespan improvement in relation to CR and CRMs is performed by Almendariz-Palacios et al. (2020). It starts with the mention of the impact of aging on pathologies, including neurodegenerative disorders, and the role of excessive calorie consumption. Next, the role of CR in the limitation of the effects of aging on the organism and reduced cellular aging is discussed: Aging is related to increased senescence in cells, leading to “key alterations (…) [which] include increased genomic instability, telomere shortening, changes in the epigenome, loss of protein homeostasis (proteostasis), deregulated nutrient sensing, mitochondrial dysfunction, stem cell exhaustion, and altered intracellular signaling” (p. 2). Mention is made of the importance of ROS in mitochondrial dysfunction, leading to cellular senescence and the role of CR on minimizing such consequences of aging is presented. The mention of the hormesis theory (preparative role of mild cellular stress leading to protection) as an explanation for the effectiveness of CR follows. The downstream effects of CR, including cellular nutrient sensing (and the related pathways, e.g., AMPK up-regulation through increased AMP/ATP ratio; mTOR down-regulation in the case of calorie restriction) are discussed as an important factor in healthy lifespan optimization. Following this, the role of amino acid restriction in healthier lifespan is presented, in relation to mTOR amino acid sensors, notably sestrin2 and cytosolic arginine sensor for mTORC1 (CASTOR), which inhibit mTOR1 in case of arginine and leucine depletion. The effects of CR and amino acid restriction are compared (similarities and differences), in terms of the involved signaling pathways. The authors then discuss CRMs (see below).

Ntsapi, C., & Loos, B. (2016). Caloric restriction and the precision-control of autophagy: A strategy for delaying neurodegenerative disease progression. *Experimental Gerontology*, *83*, 97–111. https://doi.org/10.1016/j.exger.2016.07.014

Ntsapi & Loos (2016) focus on autophagy and CR. First, estimates of people aged 60 and more by 2050 (22% worldwide) and related challenges are mentioned, notably in relation to chronic disease; including neurodegenerative diseases, linked to the accumulation of proteins. Autophagy is presented as a protective means in this respect and in reducing ROS. Autophagy can be enhanced through CR, of which the general health benefits, such as increased longevity as mentioned. Autophagy is presented as a response to nutrient deprivation with implications in neuronal homeostasis and cell survival. Three types of autophagy exist in mammals: “macroautophagy, chaperone-mediated autophagy (CMA), and microautophagy” (p. 98), which are defined and characterized through their signaling pathways. The diminishing of the autophagy types with aging is related to different processes, which can be counteracted through CR. For instance, macroautophagy is increased after already 30 minutes of nutrient depletion, whereas CMA requires 10 hours of caloric intake abstinence after depletion. CMA is preferable to macroautophagy, which is less selective in proteins degradation and could be counterproductive. The neuroprotective role of autophagy is then presented, and a distinction between macroautophagy and CMA in terms of outcomes is made. Most studies in relation to neurodegenerative diseases were interested in macroautophagy. The association of macroautophagy and CMA with different neurodegenerative diseases is discussed as well as the detrimental role of specific aggregative proteins, and the importance of mitochondrial dysfunction (which increases with age) in insufficient clearance of these proteins. The review ends with the description of the implication of targeted autophagy increased healthy lifespan, presenting IGF-1 as a central aspect in the effectiveness of calorie restriction in this process.

de Mello, N. P., Orellana, A. M., Mazucanti, C. H., de Morais Lima, G., Scavone, C., & Kawamoto, E. M. (2019). Insulin and autophagy in neurodegeneration. *Frontiers in Neuroscience*, *13*, 491. https://doi.org/10.3389/fnins.2019.00491

de Mello et al. (2019) focus on insulin and autophagy in relation to neurodegeneration. That metabolic disorders are among the most important cause of reduced life expectancy and comorbidity, including neurodegenerative diseases, is discussed first and linked to imbalanced insulin signaling, which plays an important role in neurodegeneration. How different processes such as autophagy and apoptosis, which rely upon different signaling pathways (e.g., PI3K, AMPK, Akt, mTOR), can alleviate or pejorate this is examined next. The “review summarizes the origin and the role of insulin in the CNS, and discusses the relationship between insulin and autophagy in some neurodegenerative diseases” (p. 2). Insulin resistance is presented as a factor of neurodegeneration. The effects of insulin, including on neurons are presented and brain insulin is linked to IGF-1; and insulin signaling and autophagy to neurodegeneration through different signaling pathways and several neurodegenerative diseases. CR and exercise are finally discussed as important means of regulation of autophagy.

Maharajan, N., Vijayakumar, K., Jang, C. H., & Cho, G.-W. (2020). Caloric restriction maintains stem cells through niche and regulates stem cell aging. *Journal of Molecular Medicine*, *98*, 25–37. https://doi.org/10.1007/s00109-019-01846-1

Maharajan et al. (2020) focus on the optimization of stem cells function through CR. First, mention is made of the importance of stem cells in the aging process and homeostasis in general. The review continues with mention of the presence of stem cells in niches which communicate with their environment; and next that calorie restriction and CRMs as beneficial in terms of health and longevity. Stem cell aging is defined as a decrease in their functions and list different associated impairments, which is discussed in relation to their intrinsic and extrinsic influences. The components of the niche are defined as: “cells that are physically attached to the stem cells (stromal cells and mesenchymal cells), adhesion molecules on the membrane, and membrane bound ligands or receptors in the niche cells.” (p. 28) as well as “secretory or soluble factors, such as chemokines, hormones, GF, Hedgehog, TNF, nuclear factor-kappa B (NF-kB), Wnt3, EGF, and Notch, which are produced from stem cells, stem cell progenitor cells, or niche cells.” (p. 29). How the niche affects the stem cell, for instance how the alteration of some components of the niche is detrimental to the stem cell, is discussed with examples in different organs, notably the brain. Then the regulation of the niche by non-cellular components is presented: the extracellular matrix and the importance of integrins, which notably promote the stem cells adhesion to the extracellular matrix, of which deteriorated signaling is involved in neurodegeneration. Following this, how aging is affected by energy metabolism and CR, through the activation or inhibition of different pathways (e.g., AMPK, SIRT, mTOR) is presented with the mention of normal caloric intake in relation to lower self-renewal of stem cells and anomalous functioning, but higher differentiation of stem cells; and opposite effects of CR. This differentiated impact of the presence or absence of abundant nutrients and the pathways involved are discussed. CR and CRMs are finally presented as a means to increase healthy lifespan notably through improved stem cell niche and stem cell proliferation.

Zhang, L., Xu, H., Ding, N., Li, X., Chen, X., & Chen, Z. (2021) Beneficial effects on brain micro-environment by caloric restriction in alleviating neurodegenerative diseases and brain aging. *Frontiers in Physiolology, 12*, 715443. https://doi.org/10.3389/fphys.2021.715443

Zhang et al. (2021) examine the optimization of brain extracellular microenvironment through CR, with a focus on minimizing brain aging and symptoms of neurodegenerative diseases. First, mention is made of the consequences of aging at the cellular level (e.g., cellular senescence, with disrupted gene expression) and in the brain (e.g., neuronal damage). Then the importance of the extracellular matrix and cerebrospinal fluid to the health of neurons and its broader consequences for the organism (e.g., neurodegeneration) is presented, followed by review of the signaling pathways linked with cerebral inflammation and the role of cytokines (inflammation) and microglial cells (sensing and cytokine secretion notably) in this process. The detrimental effect of metabolic waste in the extracellular microenvironment and the reduction of antioxidants that accompanies aging are presented, as well as the clearing of waste through cellular autophagy and the transport of waste outside the brain through the BBB. Next untimely removal of metabolic waste in aging due to the disruption of the permeability of the BBB are discussed. The subsequent topics addressed are the modulation of gene expression which is impacted by aging through alterations of chromosome structures; and how the cellular micro-environment can be enhanced through CR, in relation to diminished risks of neurodegenerative diseases. Implications of CR with respect to the cellular microenvironment at the molecular level (e.g., inhibition of mTOR, activation of SIRT, up-regulation of CREB leading to the increase in BDNF) are examined before the presentation of the increase in neurotrophic factor concentration as a consequence of CR and the role of CR in improving the micro-environment at the cellular level, distinguishing between mitochondrial biogenesis and autophagy, and gut microbiota. Implications of CR at the tissue level are provided, with mention of the reduction of tissue inflammation through CR. The influence of CR on the permeability of the BBB, synaptic plasticity, neurogenesis and neuron immunity are then addressed. CR interventions are the focus of the next section: a reduction in aging is induced by CR due to neurogenesis. Implications for specific diseases such as Parkinson’s and Huntington are discussed.

Yu, Q., Zou, L., Kong, Z., & Yang, L. (2020b). Cognitive impact of calorie restriction: A narrative review. *Journal of the American Medical Directors Association*, *21*, 1394–1401. https://doi.org/10.1016/j.jamda.2020.05.047

Yu and colleagues (2020b) discuss the restauration and maintenance of cognitive function through CR. Six dimensions of cognitive abilities decline with aging. The social and economic costs of such decline as well as its pharmacological treatment are presented. CR is discussed as an alternative method of dealing with cognitive decline. Next, the amelioration of a variety of aging-related health conditions through CR is detailed, and the potential mechanisms relating each time CR to cognitive function are presented: oxidative stress (areas related to cognition are more at risk of ROS damage), inflammation (secretion of inflammatory molecules by senescent cells, and their proinflammatory environment), neurogenesis and synaptic plasticity (neurotrophic factors and BDNF signaling), and neuroprotection (preservation of gray and white matter). In conclusion, a call is made for further studies to investigate several aspects of CR in order to optimize its benefits in relation to the duration of CR, the amount of CR, and the combination of CR with other interventions.

**Calorie restriction mimetics (CRMs)**

Almendariz-Palacios, C., Mousseau, D. D., Eskiw, C. H., & Gillespie, Z. E. (2020). Still living better through chemistry: An update on caloric restriction and caloric restriction mimetics as tools to promote health and lifespan. *International Journal of Molecular Sciences, 21*, 9220. doi:10.3390/ijms21239220

The summary of Almendariz-Palacios et al. (2020) is now continued in relation to CRMs. CRMs are portrayed as a means to promote healthy lifespan: Rapamycin is an immunosuppressant and antitumor medication, related to decreased mTOR1 signaling. Mention is made of its benefits in terms of lifespan extension and reduced risk of cancer as well as the augmentation of autophagy and neuroprotection among other benefits. Rapamycin analogs are then discussed. Resveratrol exerts its effects through AMPK and SIRT activation, but can have detrimental effects in high concentration. Metformin, a type 2 diabetes medication, is also discussed along with the pathways it regulates.

Bonkowski, M. S., & Sinclair, D. A. (2016). Slowing ageing by design: The rise of NAD+ and sirtuin-activating compounds. *Nature Reviews Molecular Cell Biology*, *17*, 679–690. https://doi.org/10.1038/nrm.2016.93

Bonkowski & Sinclair (2016) focus on NAD+ precursors and other SIRT activators. First, the importance of disease prevention – including neurodegeneration, and implications for longevity extension are presented. Several signaling pathways conserved from simple to complex organisms are examined next. These explain part of the effectiveness of calorie restriction in healthy aging, including AMPK, SIRTs and mTOR regulation. Then, natural molecules that are capable of activating the mentioned pathways are discussed. The review focuses on SIRTs and their activators (STACs), including NAD+ precursors. Animal models, primate and human studies are discussed in the presentation of the mechanisms of STACs. Following this, the specifics of NAD+ precursors are discussed, as NAD+ is required for the activity of SIRTs. “NAD-boosting molecules constitute a newer class of STACs gaining attention as a way to restore NAD+ levels in elderly individuals and potentially activate all seven sirtuins with a single compound. “ (Bonkowski & Sinclair, 2016, p. 17).

Iside, C., Scafuro, M., Nebbioso, A., & Altucci, L. (2020). SIRT1 activation by natural phytochemicals: an overview. *Frontiers in Pharmacology, 11,* 1225.

Iside et al. (2020) focus on the activation of SIRT1 by phytochemicals. The epigenic modifications associated with aging are presented before the role of SIRTs in reducing such consequences, and in improving a range of parameters that relate to better health, including neuroprotection. The review continues with the importance of diet in health, including the protective nature of phytochemicals, like “polyphenolic substances such as resveratrol, quercetin, curcumin, and fisetin, and of natural non-polyphenolic substances such as berberine” (p. 2). These molecules are related to the expression and activity of SIRTs. Studies which exemplify the activation of SIRTs by resveratrol, quercetin, berberine, curcumin and fisetin are reviewed, focusing on the signaling pathways involved. The conclusion focuses on the activation of SIRTs through natural CRMs, which might explain the role of these molecules in preserving or improving health, and the bioavailability of the mentioned phytochemicals.

Hofer, S. J., Davinelli, S., Bergmann, M., Scapagnini, G., & Madeo, F. (2021). Caloric restriction mimetics in nutrition and clinical trials. *Frontiers in Nutrition*, *8*, 717343. https://doi.org/10.3389/fnut.2021.717343

Hofer and colleagues (2021) focus on the availability of CRMs in nutrition. Although nutrition is essential in health, there is a relative lack of agreement regarding the definition of a healthy diet. CR is presented as a point of consensus in this debate. CRMs are defined as “pharmacologically active substances that mimic some of CR’s myriads of effects. At the core of the CRM definition, we and others argue that potential CR-mimicking compounds should in principle increase life- and/or healthspan and ameliorate age-associated diseases in model organisms, thus often the simultaneous use of the term ‘anti-aging substances’ ” (p. 2). A table of the sources of nutritional CRMs is provided, with several classes: glycolysis inhibitors, hydroxycitric acid, NAD+ precursors, polyamines, polyphenols, salicylic acid. Example of studies for each of these classes of CRMs are examined. The conclusion elaborates on the need for further studies examining points of uncertainty and confirming the beneficial effects of some CRMs in humans.

Moosavi, F., Hosseini, R., Saso, L., & Firuzi, O. (2016). Modulation of neurotrophic signaling pathways by polyphenols. Drug design, *Development and Therapy, 10*, 23-42. doi: 10.2147/DDDT.S96936

Moosavi et al. (2016) focus on the neurotrophic actions of polyphenols. First, the sources and health protective role of polyphenols in relation to different diseases - including neurodegenerative ones is presented, distinguishing between different neurotrophic functions of polyphenols (e.g., neuroprotection, neuronal proliferation, anti-oxidant effect). Details are provided on: the signaling pathways (e.g., Trk, mTOR, ERK, PI3K, CREB, Nrf2) that are targeted by different polyphenols (e.g., curcumin, quercetin, EGCG, carnosic acid) and the neurotrophic factors that are affected (BDNF, nerve growth factor – NGF, glial cell line-derived neurotrophic factor), and the functions that are achieved (e.g., antidepressant-like effects, cognition enhancement, attenuation of neuronal degeneration).

Oluwole, O., Fernando, W. B., Lumanlan, J., Ademuyiwa, O., & Jayasena, V. (2022). Role of phenolic acid, tannins, stilbenes, lignans and flavonoids in human health – a review. *International Journal of Food Science & Technology*, *57*(10), 6326–6335. https://doi.org/10.1111/ijfs.15936

Oluwole et al. (2022) focuses on the role of different polyphenol (sub)classes: tannins, phenolic acid, lignans, flavonoids and stilbenes. The classification of the large variety of polyphenols (flavonoid vs non-flavonoid) depends on the quantity of phenols their molecule contains. The functions and uses of the mentioned (sub)classes are summarized. Phenolic acids are defined, and their biological functions are mentioned: antioxidant (e.g., ROS scavenging), gastroprotective, antidiabetic, cardioprotective, chemo-preventive (e.g., lipid and protein metabolism), antimicrobial, anti-inflammatory. Their role in the prevention of neurodegeneration and neuronal injury is detailed. Next, tannins are presented, with their antimicrobial, antiviral, anti-mutagenic properties as well as their role in diabetes. Stilbenes are discussed, with mention of their chemo-preventive properties (e.g., antioxidant and anti-inflammatory) and their role in obesity and diabetes as well as the prevention of neurodegeneration. After this, the discussion continues with lignans, with chemo-preventive (e.g., hormone regulation, inhibition of cell growth), cardioprotective, antimicrobial properties. Finally, flavonoids are defined their biological functions (e.g., antioxidant, regulation of hydroperoxides), with mention of their properties: inhibition of lipid peroxidation, chemo-prevention (e.g., protective role against cancer), antimicrobial and anti-viral protection, cardioprotection, hepatoprotection, anti-inflammation and anti-neurodegeneration.

Rendeiro, C., Rhodes, J. S., & Spencer, J. P. (2015). The mechanisms of action of flavonoids in the brain: Direct versus indirect effects. *Neurochemistry international, 89*, 126-139. https://doi.org/10.1016/j.neuint.2015.08.002

Rendeiro et al., (2015) examine the mechanisms of action of flavonoid polyphenols in the context of the prevention of neurodegenerative diseases. The elevation of serum nitric oxide is one of the benefits of flavonoids. The bioavailability of flavonoids metabolites in the brain is mentioned in relation to their ability to cross the BBB. Several flavonoid metabolites have been detected in the brain, which supports such requirement for neuroprotection. Human cognition is influenced by flavonoids and reported benefits of several groups of flavonoids are mentioned. The mechanisms of action of flavonoids, i.e., the modulation of synaptic plasticity and the pathways of such action are discussed, including the indirect mechanisms which relate to the improvement of the neurovascular system and cerebrovascular functioning (e.g., blood oxygenation levels, increased brain vascularization), through increased nitric oxide bioavailability notably. The correlational nature of the reported association is discussed with the need for research that improves the mechanistical assumptions relying upon strong empirical evidence, some of which is already available. That neither long term effects of flavonoids have yet been thoroughly investigated in humans, nor their recommended daily dose is also discussed.

Zhang, L.-X., Li, C.-X., Kakar, M. U., Khan, M. S., Wu, P.-F., Amir, R. M., Dai, D.-F., Naveed, M., Li, Q.-Y., Saeed, M., Shen, J.-Q., Rajput, S. A., & Li, J.-H. (2021). Resveratrol (RV): A pharmacological review and call for further research. *Biomedicine & Pharmacotherapy*, *143*, 112164. https://doi.org/10.1016/j.biopha.2021.112164

Zhang et al. (2021) focus on the pharmacological actions of resveratrol, a stilbenoid polyphenol. First a brief presentation of resveratrol is proposed with a list of its functions (e.g., cardioprotection, reduction of neurotoxicity) and a brief mention of the signaling pathways involved (e.g., reduction of oxidative stress, improvement of mitochondrial health). Next, the chemical structure of resveratrol is presented, with mention of most important nutritional sources (e.g., tomatoes, peanuts, red grapes, different berries, pomegranates, cranberries, dark chocolate) and intake recommendations. The presentation continues with the pharmacokinetics of resveratrol including its metabolization in the liver, its plasma concentration and its distribution in different organs. Its mechanisms of action are explained, focusing in more detail on the signaling pathways of which it supports the regulation. Finally, a health perspective on resveratrol is proposed in the form of a synthesis, focusing on its antioxidant and anti-inflammatory actions as well as its role in protection against neurodegenerative diseases among others.

Moradi, S. Z., Jalili, F., Farhadian, N., Joshi, T., Wang, M., Zou, L., Cao, H., Farzaei, M. H., & Xiao, J. (2022). Polyphenols and neurodegenerative diseases: Focus on neuronal regeneration. *Critical Reviews in Food Science and Nutrition*, *62*(13), 3421–3436. https://doi.org/10.1080/10408398.2020.1865870

Moradi et al. (2022) focus on neuronal regeneration following polyphenol intake. Following the mention of the scarcity of organ donation and the importance of regenerative medicine, the review continues with explanations of nervous system diseases (such as AD, PD, HD, ALS) and their therapy, notably focusing on cytokines, growth factors, neurotrophic factors and stem cells. Next, the therapeutic effects of polyphenols in neurodegenerative disorders are examined, distinguishing between flavonoids, isoflavones, phenolic acids and tannins as classes of polyphenols for which such benefits were investigated. The mechanisms of action are detailed, such as ROS scavenging, reduced beta-amyloid accumulation, regulation of apoptosis and gene expression. Example of studies on the regenerative effect of polyphenols are proposed, focusing on a single or multiple molecules. Then the role of stem cells in neuronal regeneration is examined: “Stem cell therapy is one of the most important branches of regeneration medicine that has enhanced the researcher’s hope to treat or improve the condition of the patient with varying debilitating diseases” (p. 4). Examples of studies are provided. Neuronal regeneration through chemical stimulation and the emerging role of gene therapy are presented. A neuronal perspective then addresses the role of polyphenols in regeneration. The antioxidative properties of polyphenols, the potential improvements in neurogenesis, neuronal connectivity, nerve damage, neuron survival notably stemming from of a variety of polyphenols are presented next. Finally, challenges and future perspectives in the field are discussed and the potential of the mentioned treatments in alleviating consequences of aging is highlighted.

Grewal, A. K., Singh, T. G., Sharma, D., Sharma, V., Singh, M., Rahman, Md. H., Najda, A., Walasek-Janusz, M., Kamel, M., Albadrani, G. M., Akhtar, M. F., Saleem, A., & Abdel-Daim, M. M. (2021). Mechanistic insights and perspectives involved in neuroprotective action of quercetin. *Biomedicine & Pharmacotherapy*, *140*, 111729. https://doi.org/10.1016/j.biopha.2021.111729

Grewal et al. (2021) delineate the mechanisms of neuroprotection of quercetin, a flavonoid polyphenol. The neuroprotective effect of polyphenols (notably reduced oxidative damage and reduction in risks for several neurodegenerative diseases) is examined. Nutritional sources of quercetin (e.g., red onion, garlic, apple, pomegranate), its chemical formula and pharmacological action are presented. The neuroprotective effect of quercetin can be enhanced through nano-emulsion. The pathogenesis of neurodegenerative diseases is discussed. It notably involves mitochondrial dysfunction, oxidative stress, elevated apoptosis, and inflammation. The review elaborates on different neurodegenerative diseases for which quercetin has been shown to reduce risks and symptoms and proposed mechanisms of action (e.g., for Alzheimer’s disease: improvement in neuroinflammation, oxidative stress, amyloid plaques cholinesterases, notably; for Parkinson Disease: improvement in neuroinflammation, oxidative stress, apoptosis, autophagy, notably; for Huntington disease, improvement in neuroinflammation, oxidative stress, cognitive deficits, neuronal dysfunction, notably). Finally, the signaling pathways influenced by quercetin are detailed, among others: paraoxonase 2 (PON2), the nuclear factor erythroid 2-related factor 2 (Nrf2) - Adenylate/uridylate-rich elements (ARE).

Ravula, A. R., Teegala, S. B., Kalakotla, S., Pasangulapati, J. P., Perumal, V., & Boyina, H. K. (2021). Fisetin, potential flavonoid with multifarious targets for treating neurological disorders: An updated review. *European Journal of Pharmacology*, *910*, 174492. https://doi.org/10.1016/j.ejphar.2021.174492

Ravula et al. (2021) provide an updated review on the neuroprotective and neurogenerative impact of the flavonoid polyphenol fisetin. Neurological diseases are a leading cause of disability and mortality and the difficulty of therapy of such diseases is discussed. The complementary role of flavonoids in the treatment of neurodegenerative disorders is presented with mention of the “potential of flavonoids as antioxidants, antiviral, anti-inflammatory, anti-carcinogenic, anti-bacterial, neurotrophic, neuroprotective, and immune-stimulants” (p. 2). The functions of fisetin in particular in the prevention of neurodegeneration are mentioned: reduced neuroinflammation, improved immune response, and the optimization of signaling pathways that are disrupted by aging and in neurodegenerative disorders. Research showing that fisetin is effective in improving cognition is presented, which is followed by the mechanisms affected by fisetin, some of which were not discussed in the main text of our review, such as: the ‘modulation of Cdk5/p35’ (associated with neuroinflammation, synaptic damage notably and neuronal death), the ‘regulation of eicosanoids’ (pro-inflammatory lipid componds), ‘saving ATPase’ (important in homeostasis, impaired by oxidative stress), ‘fisetin-TFEB—MTORC1-Nrf2 linkage’ (role in autophagy notably), the ‘modulation of Kelch-like ECH-associated protein 1 (Keap 1)- nuclear factor erythroid 2-related factor 2 (Nrf2)- antioxidant response elements (ARE) pathway’ (control of xenobiotic damage and oxidative stress; maintenance of enegery metabolism and redox balance in the cells), ‘Regulation of advanced glycation end products (AGEs)’ (inhibit protein functions, impair antioxidant enzymes, aggregate proteins, notably) ; as well as the ‘modulation of CREB’ (involved in learning and memory), ‘NAD+ degradation’ (see above), the ‘restauration of synaptic proteins’, the ‘elevation of Acetyl CoA’ (involved in glycolysis and fatty acid synthesis & oxidation, abnormalities in Acetyl-CoA can lead to dementia, precursor to neurotransmitter Acetylcholine), and the ‘modulation of NF-kB’ (pro-inflammatory, regulates cytokine expression, involved in neurodegeneration). Research on fisetin in relation to neurodegeneration is summarized in Table 1. Figure 7 indicates that fisetin decreases or inhibits among others: lipid peroxides, IL-6, TNF-alpha, NF-kB, nitric oxide, superoxides, serum homocysteine; and increases or activates among others: BDNF, acetylcholine, butyrylcholine, serotonine, noradrenaline, Nrf2, TrkB, MEK-ERK.

**Other nutrients inducing neurotrophic effects**

Several molecules which are not considered CRMs have neurotrophic effects. Some of these molecules are briefly introduced in the continuation of this annotated bibliography.

Heberden, C. (2016). Modulating adult neurogenesis through dietary interventions. *Nutrition Research Reviews*, *29*, 163–171.

The modulation of adult neurogenesis with polyphenols and n-3 polyunsaturated fatty acids is examined in Heberden et al. (2016). The potential of the human brain to regenerate might be less than that of rodents and limited to specific areas with neural stem cells, which are presented: the subgranular zone of the dentate gyrus, the subventricular zone of the lateral ventricle, and the median eminence of the hypothalamus. The regulators and effectors of neurogenesis (through neurotrophins and growth factors) are examined with mention of the concentrated nature of neural stem cells niches. The role of leptin as a promoter of neural stem cell proliferation, as well as other peptide hormones, is highlighted. The detrimental effect of aging on neurogenesis is examined, following with dietary influences on this process. The unfavorable effects of overnutrition are addressed, notably in terms of proliferation and survival of new neurons. The potential of dietary intervention through polyphenols (see also above) and n-3 polyunsaturated fatty acids is addressed: beneficial in terms of neural stem cell proliferation and differentiation, neurogenesis, and arborization of new neurons. The targets and signaling pathways involved are detailed, such as AMPK, BDNF/CREB, NF-kB, SIRT1. The gut microbiota is presented as a potential effector of nutrition induced adult neurogenesis, for instance through their action on metabolism and inflammation.

Carneiro, S. M., Oliveira, M. B. P., & Alves, R. C. (2021). Neuroprotective properties of coffee: An update. *Trends in Food Science & Technology, 113,* 167-179. https://doi.org/10.1016/j.tifs.2021.04.052

Carneiro et al. (2021) provide an updated systematic literature review on the neuroprotective effects of coffee. The stimulant properties in the CNS are presented as a factor contributing to the large worldwide consumption of coffee. The health benefits of coffee are also mentioned. The aims of the review are to synthesize the literature relating coffee consumption to the reduction of the risk of neurodegenerative disorders and the mechanisms involved. There are more than 1,000 components in coffee of which the most important are presented. The most studied, caffeine is antagonist of the inhibitory neurotransmitter adenosine, which notably explains the stimulant properties of coffee. Another important compound is trigonelline, which is related to neuroprotection. The neurotransmitter serotonin is also a compound of coffee. Coffee also contains polysaccharides, which have an impact on the gut microbiome that enhances cognitive function and the anti-oxidants cafestol and kahweol. Nicotinic acid (B3) is a component of roasted coffee. Other components are enumerated and their health benefits mentioned (e.g., antioxidant, anti-inflammatory, anticarcinogenic). Next, the role of coffee in the prevention of several neurodegenerative diseases is examined: for instance, through the reduction of excitotoxicity, oxidation and inflammation (caffeine), or the prevention of memory impairments by trigonelline. The phenolic components and other components of coffee and their neuroprotective properties are also mentioned and research on the protective role of coffee in the risk for different neurodegenerative diseases is detailed. The positive global health impact of coffee, notably in relation to neurodegenerative disorders, is discussed.

Chen, X., Ghribi, O., & Geiger, J. D. (2010). Caffeine protects against disruptions of the blood-brain barrier in animal models of Alzheimer’s and Parkinson’s diseases. *Journal of Alzheimer’s Disease*, *20*(s1), S127–S141. https://doi.org/10.3233/JAD-2010-1376

Chen et al. (2010) focus on the protective role of caffeine with regards to the integrity of the BBB, which is one of the neuroprotective effects of caffeine. The function of the BBB (to isolate the brain from toxins, notably) and the structure of the BBB are first described. The disruption of the BBB is mentioned as one of the causes of the onset of AD (notably as a result of high cholesterol levels), and potentially PD (BBB disruption occurring before the loss of dopaminergic neurons, characteristic of PD). Three mechanisms through which caffeine is believed to protect the BBB are examined: notably by “blocking cell surface adenosine receptors, through inhibition of cAMP phosphodiesterase (PDE) activity, and by affecting the release of calcium from intracellular stores” (p. S131). Next, the integrity of the BBB through chronic caffeine consumption might explain part of the neuroprotective effect of caffeine, and examples relating for instance to better memory and cognition, reduced loss of dopaminergic neurons, as indicators of such effect are provided. The protective actions of caffeine on the BBB are mentioned as primary in comparison to neuroprotective actions of caffeine.

Cui, X., Gooch, H., Petty, A., McGrath, J. J., & Eyles, D. (2017). Vitamin D and the brain: Genomic and non-genomic actions. *Molecular and Cellular Endocrinology, 453,* 131-143. https://doi.org/10.1016/j.mce.2017.05.035

Cui et al. (2017) examine the role of vitamin D in brain development, neuroprotection and immunity. Vitamin D is presented as a developmental neurosteroid, positively involved in brain development and neuroprotection. The signaling functions of vitamin D through the Vitamin D Receptor (VDR) are detailed. Brain morphology and physiology are importantly linked to vitamin D. The goals of the review are presented: the examination of the actions of vitamin D in “brain cell differentiation, neurotransmitter release, and calcium signaling via its genomic and non-genomic functions.” (p. 132). VDR is distributed in the brain. Rodent models are discussed first as (“[the] pattern of VDR distribution indicates that vitamin D may be involved in the proliferation and/or differentiation of neuronal stem cells”, p. 132), then the human brain as (“[the] VDR protein was also identified in the human brain and the distribution pattern of the VDR was found to be strikingly similar to that reported in rodents, p. 133). The genomic actions of vitamin D are addressed, in particular its involvement in apoptosis, cell proliferation, and neural growth. The role of vitamin D in the regulation of the development of dopaminergic neurons and other cells, and then the differentiation of adult neural stem cells and myelination is also examined. The effect of vitamin D on neuronal survival, programmed glioma cell death and neurotransmitter release is addressed. Which is followed by the neuroprotective effects of vitamin D in the aged brain and its relation to neurotrophic factors, BDNF notably. Next, mention is made that vitamin D can compensate neuroinflammation in the aging brain, as well as immune response. Also, “vitamin D could potentially increase local estrogen synthesis in glial cells, which is important to maintain neuronal function” (p. 137). The non-genomic functions of vitamin D, an underexplored area, are then examined, this includes: the role of vitamin D in the regulation of calcium and kinase activated pathway signaling recently discovered in the developing cortex, and other modulatory effects of vitamin D, which for some have neuroprotective properties. The therapeutic potential of vitamin D, and the negative consequences of deficiencies in vitamin D are finally discussed.
